# FUS Phase Separation Is Modulated by a Molecular Chaperone and Methylation of Arginine Cation-π Interactions

**DOI:** 10.1016/j.cell.2018.03.056

**Published:** 2018-04-19

**Authors:** Seema Qamar, GuoZhen Wang, Suzanne J. Randle, Francesco Simone Ruggeri, Juan A. Varela, Julie Qiaojin Lin, Emma C. Phillips, Akinori Miyashita, Declan Williams, Florian Ströhl, William Meadows, Rodylyn Ferry, Victoria J. Dardov, Gian G. Tartaglia, Lindsay A. Farrer, Gabriele S. Kaminski Schierle, Clemens F. Kaminski, Christine E. Holt, Paul E. Fraser, Gerold Schmitt-Ulms, David Klenerman, Tuomas Knowles, Michele Vendruscolo, Peter St George-Hyslop

**Affiliations:** 1 Cambridge Institute for Medical Research, Department of Clinical Neurosciences, University of Cambridge, Cambridge CB2 0XY, UK; 2 Department of Chemistry, University of Cambridge, Cambridge CB2 1EW, UK; 3 Department of Physiology, Development, and Neuroscience, University of Cambridge, Cambridge CB2 3DY, UK; 4 Tanz Centre for Research in Neurodegenerative Diseases and Departments of Medicine, Medical Biophysics and Laboratory Medicine and Pathobiology, University of Toronto, Toronto, ON M5S 3H2, Canada; 5 Department of Chemical Engineering and Biotechnology, University of Cambridge, Cambridge CB3 0AS, UK; 6 Department of Biomedical Sciences, Cedars Sinai Medical Center, Los Angeles, CA 90048, USA; 7 Centre for Genomic Regulation, the Barcelona Institute for Science and Technology, 08003 Barcelona, Spain; 8 Universitat Pompeu Fabra, 08003 Barcelona, Spain; 9 Institució Catalana de Recerca i Estudis Avançats, 08010 Barcelona, Spain; 10 Departments of Medicine, Neurology, and Ophthalmology and Departments of Epidemiology and Biostatistics, Boston University, Boston, MA 02118, USA; 11 Cavendish Laboratory, Department of Physics, University of Cambridge, Cambridge CB3 0HE, UK

**Keywords:** phase separation, neuronal ribonucleoprotein granule, synaptic new protein synthesis, membraneless organelle, cation-π, arginine methylation, citrullination, frontotemporal dementia, AFM-IR, phase-sensitive fluorescent dyes

## Abstract

Reversible phase separation underpins the role of FUS in ribonucleoprotein granules and other membrane-free organelles and is, in part, driven by the intrinsically disordered low-complexity (LC) domain of FUS. Here, we report that cooperative cation-π interactions between tyrosines in the LC domain and arginines in structured C-terminal domains also contribute to phase separation. These interactions are modulated by post-translational arginine methylation, wherein arginine hypomethylation strongly promotes phase separation and gelation. Indeed, significant hypomethylation, which occurs in FUS-associated frontotemporal lobar degeneration (FTLD), induces FUS condensation into stable intermolecular β-sheet-rich hydrogels that disrupt RNP granule function and impair new protein synthesis in neuron terminals. We show that transportin acts as a physiological molecular chaperone of FUS in neuron terminals, reducing phase separation and gelation of methylated and hypomethylated FUS and rescuing protein synthesis. These results demonstrate how FUS condensation is physiologically regulated and how perturbations in these mechanisms can lead to disease.

## Highlights

• Intermolecular β sheet hydrogen bonding and cation-π interactions drive FUS demixing
• Cation-π interactions form between C-terminal arginines and N-terminal tyrosines
• Cation-π strength is regulated by arginine methylation and interacting proteins
• FUS hypomethylation in FTLD induces FUS gelation and impairs RNP granule function

## Graphical Abstract

**Figure:**
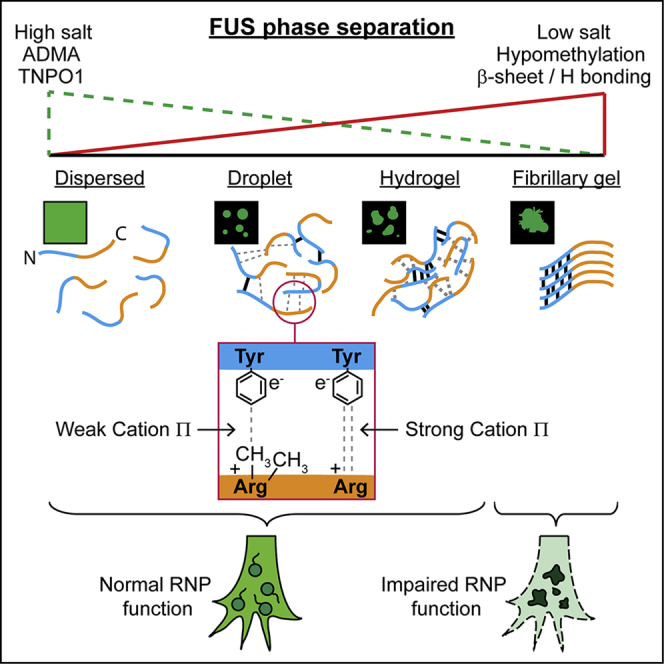

## Teaser

Phase transition of the RNA-binding protein FUS is mediated by cation-π interactions between C-terminal arginines and N-terminal tyrosines and is modulated by arginine methlylation.

## Introduction

**Figure 1:**
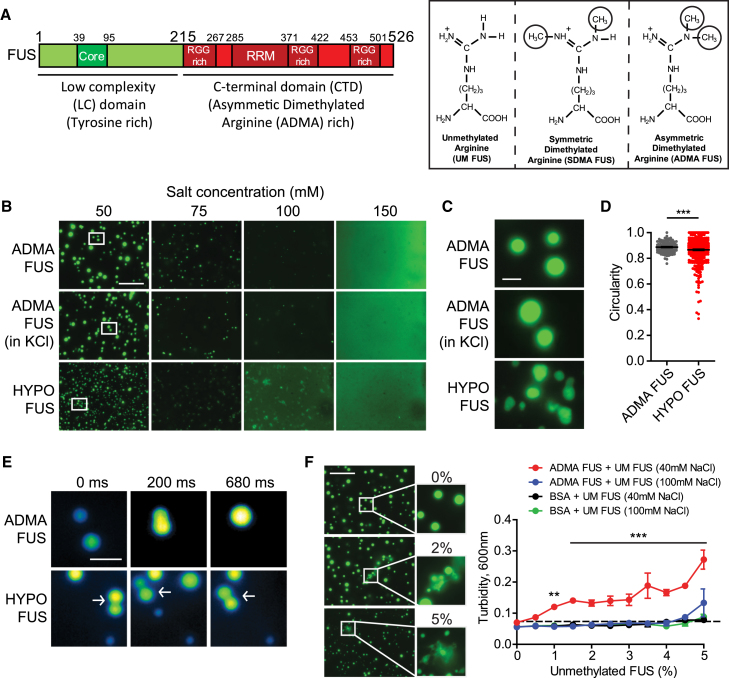
Phase Separation of Full-Length FUS at Physiological Temperature and Protein Concentration Is Modulated by Salt Concentration and Arginine Methylation (A) Left: Schematic of domain architecture and location of tyrosine-rich and arginine-rich domains. Right: Arginine methylation species. RRM, RNA recognition motif; RRG, arginine glycine-rich domain. (B) Salt-dependent phase separation of ADMA FUS and HYPO FUS. Top*:* Representative images of phase separation of 1 μM EmGFP-tagged ADMA FUS in 50–150 mM NaCl. At 150 mM NaCl, ADMA FUS is mono-dispersed, but phase separates into spherical droplets at lower salt concentrations. Middle*:* Representative images of ADMA FUS for KCl concentrations of 50–150 mM. Bottom: Identically prepared HYPO FUS phase separates at higher salt concentrations (100 mM) into small, irregularly shaped condensates. White boxes indicate location of magnified images in (C). Scale bar, 25 μm. (C) High-magnification images of condensates from (B). Scale bar, 5 μm. (D) Quantitative analysis of sphericity: ADMA FUS condensates (gray) in 50 mM NaCl are spherical. HYPO FUS condensates (red) are less spherical (t = 3.47, p = 0.0006). n ≥ 121 particles/FUS subtype; n > 3 independent replications. Error bars, SEM. (E) Sequential structured illumination microscopy images of individual droplet collisions at 0, 200, and 680 ms. ADMA FUS (top) fuse. HYPO FUS collide but do not fuse. Scale bar, 2 μm. See Video S1 (ADMA FUS) and Video S2 (HYPO FUS). (F) Representative images and quantitative turbidity graphs of phase separation arising from mixing EmGFP-tagged ADMA FUS with the indicated percentage of fully unmethylated UM FUS. Preparations containing > 1% UM FUS form small non-spherical, non-fusing, and amorphous assemblies. Scale bar, 25 μm. Two-way ANOVA with Bonferroni post hoc test versus ADMA FUS at 40 mM, n ≥ 3 replications, ^∗∗^p < 0.01, ^∗∗∗^p < 0.001. Error bars, SEM. See also Figure S1 and Videos S1 and S2.

Fused in sarcoma (FUS) is an RNA-binding protein involved in RNA transcription, splicing, transport, and translation. FUS undergoes rapid, physiologically reversible phase separation between dispersed, liquid droplet, and hydrogel states (Han et al., 2012, Kato et al., 2012, Murakami et al., 2015, Patel et al., 2015). The droplet and hydrogel states are stabilized by hydrogen bonding between antiparallel β sheet motifs formed by core residues 39–95 in the low-complexity (LC) domain (Murray et al., 2017) (Figure 1A). The ability of FUS and other proteins with intrinsically disordered domains to undergo phase separation likely contributes to the role of FUS in forming transient membrane-free organelles, such as ribonucleoprotein (RNP) granules (Weber and Brangwynne, 2012). These dynamic structures take up, sequester, transport, and then release key RNA and protein cargos that regulate local RNA and protein metabolism in subcellular niches, such as axon terminals and dendrites (Holt and Schuman, 2013, Sephton and Yu, 2015). When these processes go awry (e.g., because of pathogenic missense mutations), they trigger disease, such as familial amyotrophic lateral sclerosis (fALS) and frontotemporal lobar degeneration (FTLD).

Given the crucial role of intrinsically disordered proteins like FUS in multiple fundamental biological processes, understanding the molecular and cellular factors that control their reversible condensation would be invaluable. This knowledge could also yield avenues for therapeutic intervention in diseases associated with aberrant assembly of these proteins, such as FUS-associated ALS (fALS-FUS) and frontotemporal lobar degeneration (FTLD-FUS).

A potentially powerful clue to the identity of such factors is the observation that arginines in FUS, which are predominantly located in the structured C-terminal domain (sCTD), are normally heavily methylated as mono- or dimethylated forms (Figure 1A) (Rappsilber et al., 2003). However, in FTLD-FUS, FUS is hypomethylated and accumulates in neurons as nuclear and cytoplasmic aggregates that frequently also contain EWS, TAF15, and transportin 1 (TNPO1) (Dormann et al., 2012, Neumann et al., 2012). These observations suggest that physiological fluctuations in its arginine methylation and/or its interactome might physiologically control FUS phase behavior.

**Figure 2:**
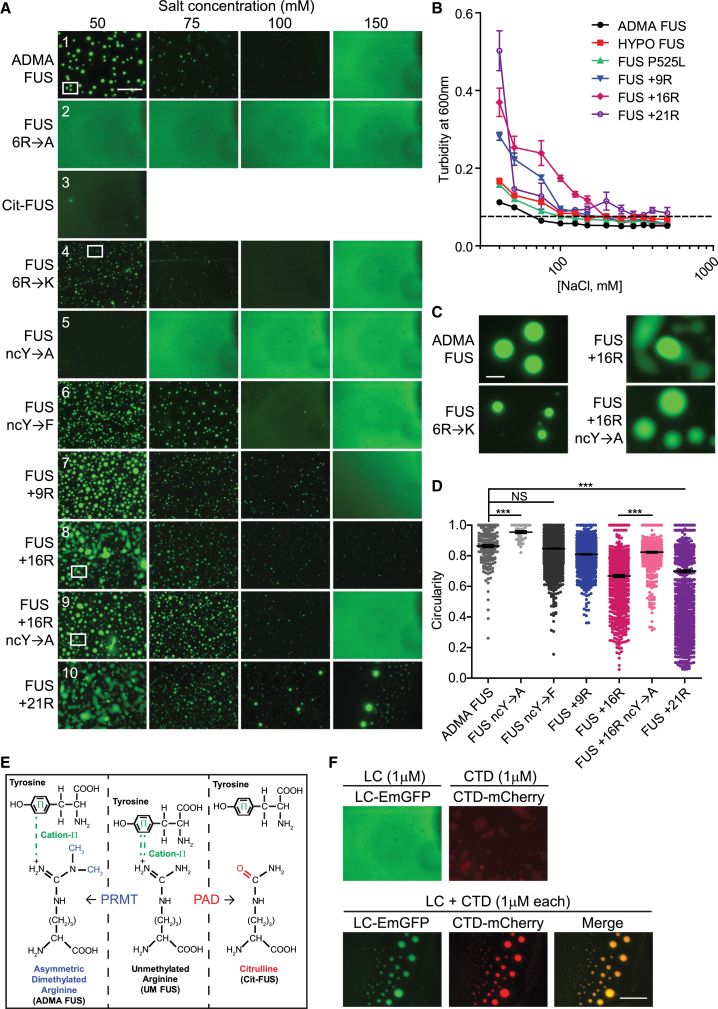
Phase Separation Is Driven by Cation-π Interactions between Arginines in RGG Motifs in the Structured C-Terminal Domain and Tyrosines near the Core of the LC Domain (ncY) For a Figure360 author presentation of Figure 2, see the figure legend at https://doi.org/10.1016/j.cell.2018.03.056. (A) The strength of cation-π interactions can be modulated by varying the number of arginine residues or by varying the number of tyrosine residues. Row 1: Representative images of phase separation by EmGFP-tagged ADMA FUS in 50 −150 mM NaCl. Row 2: Mutating arginines 216, 259, 407, 472, 473, and 476 to alanine (6R→A) abrogates phase separation. Row 3: Enzymatic conversion of arginines to citrullines abrogates phase separation. Row 4: Mutation of these arginines to lysine (6R→K) preserves phase separation. Row 5: Mutating ncYs 113, 122, 130, 136, 143, 149, 161 to alanine (ncY→A) reduces phase separation. Row 6: Mutating the same tyrosines to phenylalanine (ncY→F) preserves phase separation. Rows 7, 8, and 10: Addition of arginine residues (FUS +9R, FUS +16R, FUS +21R) permits phase separation at higher salt concentrations. Row 9: Adding ncY→A to FUS +16R (FUS +16R ncY→A) rescues phase separation (150 mM). Scale bar, 25 μm. (B) Phase separation/turbidity diagram for constructs in (A). Error bars, SEM; n ≥ 3 replications. (C) Representative images of highly spherical ADMA FUS and FUS 6R→K assemblies. The nonspherical FUS +16R droplets can be rescued by FUS +16R ncY→A mutations. (D) Circularity (sphericity) graph: ADMA FUS are spherical. Replacing ncYs with alanine (ncY→A) increases circularity, likely because there is reduced progression to gelation (Student’s t test, Satterthwaite method for unequal variances: t = 7.46, degrees of freedom [df] = 155, p = 5.91 × 10^−12^). Replacing ncYs with phenylalanine (ncY→F) supports normal phase separation (t = 1.69, df = 213, p = 0.092). Augmenting cation-π interactions increases gelation and reduces circularity (i.e., FUS +9R, FUS +16R, FUS +21R) (Student-Newman-Keuls multiple comparisons of means test: F = 64.57_[6, 6674]_, p = 2.84 × 10^−78^). Decreasing the augmented cation-π drive in FUS +16R by reducing the available near-core tyrosines (FUS +16R ncY→A) restores normal phase separation and circularity (FUS +16R-ncY→A vs. FUS+16R: t = 16.84, df = 987, p = 3.97 × 10^−56^); FUS +16R-ncY→A versus ADAM FUS: t = 3.98, df = 257, p = 8.98x10^−5^). N > 3 independent replications. Error bars, SEM. (E) Schematics of PRMT-mediated dimethylation of arginine to create ADMA FUS; PAD-mediated conversion of arginine to citrulline; and cation-π interactions between tyrosine rings and arginine guanidino side chain. (F) Tyrosine-rich LC domain and arginine-rich sCTD cooperatively support phase separation. Top row: representative images of EmGFP-LC domain (aa 1–214) alone (left) and mCherry-CTD (aa 215–526) alone (right) at 1 μM FUS, 50 mM NaCl, showing minimal phase separation. Bottom row: brief (<2 min) phase separation occurs upon mixing LC domain (green) with sCTD (red). Merged (orange). Scale bar, 25 μm. See also Figure S2. Figure360: An Author Presentation of Figure 2

To assess this possibility, we investigated the effect of manipulating (1) the number and post-translational methylation state of arginines (Figure 1A) and (2) the interactions with known FUS-binding proteins. These experiments confirm that FUS phase separation is exquisitely modulated by (1) arginine methylation state (which tunes the strength of cation-π interactions between the structured C-terminal and the disordered N-terminal domains) and (2) binding of TNPO1, which acts as a molecular chaperone in peripheral compartments of neurons. Crucially, hypomethylation of FUS promotes formation of stable condensates comprising intermolecular β-sheet-rich FUS assemblies that disrupt RNP granule function in neuronal terminals and cause FTLD-FUS.

## Results

### FUS Phase Behavior *In Vitro* Is Modulated by Salt and FUS Concentration

Prior work has established that the LC domain of FUS can form β-sheet-rich condensates when cooled at high protein concentrations (50–133 μM) in the presence of crowding agents (e.g., polyethylene glycol and dextran). However, to gain a quantitative understanding of how regions outside the LC domain might influence the phase behavior of full-length FUS, we expressed and purified wild-type, full-length human FUS from eukaryotic S*f*9 cells with or without an Emerald GFP (EmGFP) tag. We chose this system over bacterial production systems because it allows analysis of FUS that has undergone physiological eukaryotic post-translational modification. We then used this material to explore the impact of variations in temperature (4°C–37°C) and salt (50–150 mM NaCl or KCl) at physiological FUS protein concentrations (≤5 μM).

These experiments revealed that variations in temperature (4°C–37°C) had little effect on the phase state of full-length FUS at physiological concentrations (∼1 μM). In contrast, FUS phase behavior was profoundly affected by variations in the concentration of salts, such as NaCl and KCl. Specifically, at 1 μM FUS, decreasing concentrations of NaCl or KCl from 150 to 50 mM caused rapid phase separation of FUS into hundreds of small droplets (2.29 ± 0.15 μm diameter; n = 128 assemblies) (Figure 1B). These assemblies, which appeared within seconds, were approximately spherical (Figures 1C–1E), and underwent fusion events that could be monitored using structured illumination microscopy (Figure 1E; Video S1). These effects were not influenced by the presence or the absence of an EmGFP tag.

### Hypomethylation of Selected FUS Arginines Promotes Phase Separation

**Figure S1:**
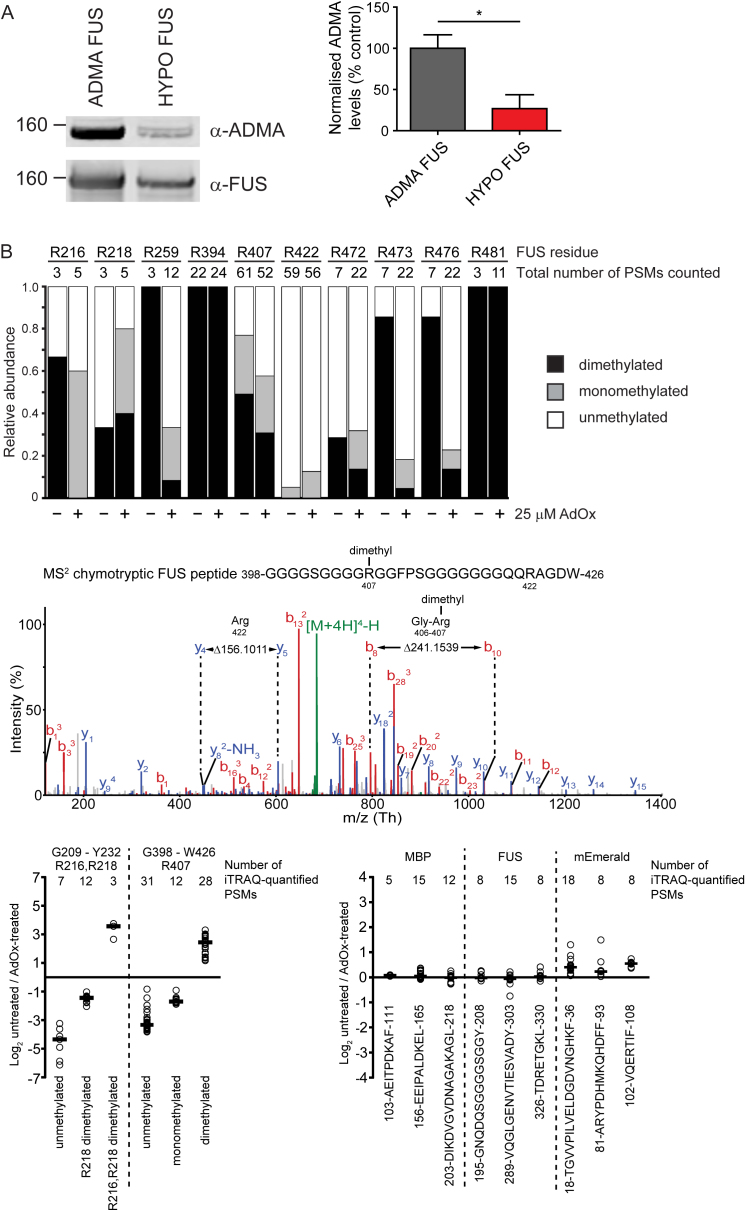
25 μM AdOx Treatment Significantly Reduces Asymmetric Dimethylation of FUS Purified from Sf9 Cells, Related to Figure 1 (A) *Left:* Representative western blot of MBP-FUS-EmGFP protein purified from Sf9 cells after 4 days of 25μM AdOx, or DMSO control. *Right:* Quantification of western blots. n = 4 per experimental group, one-tailed, Mann-Whitney U test, ^∗^p ≤ 0.05, error bars = SEM. (B) *Top:* FUS dimethylation sites differ in their apparent susceptibility to AdOx treatment. Depicted are the relative proportion of peptide-to-spectrum matches (CID MS2 spectra of unlabeled peptides identified with the PEAKS algorithm) comprising a given FUS arginine residue observed in an unmodified, mono- or dimethylated state. For most FUS arginine residues, methylation decreased in AdOx treated relative to mock treated cells, except for R394 and R481 which were consistently observed to be dimethylated. *Middle:* Orbitrap CID MS2 spectrum of a chymotryptic FUS peptide dimethylated at R407 identified with confidence exceeding 99%. The other arginine residue present (R422) was unmodified, as evidenced by a continuous y-ion series indicating no post-translational modification in this part of the peptide. The spectrum is representative of 77 peptide-to-spectrum matches for this region of FUS, which consistently identified (mono or di) methylated R407 accompanied by unmodified R422. *Bottom left:* Evidence for AdOx-dependent inhibition of arginine methylation. The graph depicts the relative ratios of unmethylated, monomethylated and dimethylated versions of the FUS peptides 209-GQQDRGGRGRGGSGGGGGGGGGGY-232 and 398-GGGGSGGGGRGGFPSGGGGGGGQQRAGDW-426 in untreated and AdOx-treated FUS preparations. Circles represent individual quantifications. The number of quantifications (based on separate peptide-to-spectrum matches) underlying each cumulative quantification are listed above the graph. Horizontal marks depict median Log2 ratios for a given peptide and modification. *Bottom right:* AdOx treatment did not affect overall abundance of peptides in untreated versus AdOx-treated samples. Abundance ratios of nine chymotryptic peptides from the MBP-FUS-EmGFP. All peptides depicted either lack arginine residues or contain arginine residues but were not observed to be methylated or dimethylated.

To explore the effects of changes in FUS methylation, we isolated full-length, wild-type human FUS, with or without an EmGFP tag, from eukaryotic Sf9 cells grown in the presence of 25 μM adenosine-2,3-dialdehyde (AdOx), a widely used inhibitor of arginine methyltransferase activity (Dormann et al., 2012). Arginine methyltransferases are components of RNP granules and therefore likely relevant to the biology of FUS phase separation (Scaramuzzino et al., 2013). Western blots of FUS protein from AdOx-treated cells showed a significant reduction in asymmetrically dimethylated FUS (ADMA FUS) (Figures 1A and S1A).

To identify which arginines were methylated and to quantitatively assess the reduction in arginine methylation induced by AdOx, we used both isobaric tags for relative and absolute quantitation (iTRAQ) and spectral counting mass spectrometry methods. In full-length FUS from untreated Sf9 cells, methylation was not homogeneously distributed across all 37 arginines (Figure S1B). At least 9 arginines were dimethylated, and these were predominantly located in glycine-rich clusters. However, several arginines were predominantly unmethylated, even when neighboring arginines were dimethylated. The effect of AdOx was also nonuniform. Thus, arginines 216, 259, 407, 473, and 476 were converted from a significantly dimethylated state to a predominantly mono- or unmethylated state. In contrast, arginines 394 and 481 remained predominantly dimethylated (Figure S1B). These differences were robust, being replicated in both the iTRAQ and the spectral counting analyses and were not altered by the presence or the absence of the EmGFP tag.

To assess the effects of reduced methylation on FUS phase behavior, we repeated the phase transition experiments on hypomethylated FUS (HYPO FUS) purified from AdOx-treated Sf9 cells. HYPO FUS condensed into many small assemblies (1.46 ± 0.11 μm), often with non-spherical shapes, fewer fusion events, and right-shifted the phase diagram in a manner similar to the effects of fALS- FUS mutations (Figures 1B, 1D, 1E, and 2B; Video S2; ADMA FUS, black; FUS P525L, green; HYPO FUS, lower red line; p < 0.0006). These differences were not influenced by the EmGFP tag.

The dramatic effect of FUS arginine methylation on phase separation raises the question of what proportion of FUS needs to be hypomethylated before significant changes in phase behavior could occur. If this proportion was small, then modulating FUS methylation might provide a physiological mechanism to dynamically change FUS assembly. Furthermore, if this process became uncontrolled, the accumulation of excessive quantities of hypomethylated FUS might then cause disease by promoting the formation of stable and biologically irreversible fibrillar hydrogel assemblies in a manner analogous to fALS-FUS mutations (Murakami et al., 2015). To address this question, we repeated the phase separation experiments at the boundary conditions of 2 μM full-length FUS and 40 mM NaCl at 23°C, but included varying quantities of fully unmethylated FUS (UM FUS) purified from *E. coli* (0.5%–5% of total FUS). These studies revealed that when UM FUS comprised more than 1% of total FUS there was (1) increased formation of small non-spherical, non-fusing assemblies and (2) the appearance of larger diffuse assemblies that are likely fibrillary hydrogel condensates (Figure 1F, middle and lower panels, p < 0.01). These results indicate that even small quantities of unmethylated FUS (< 5%) could induce transition of dispersed FUS into liquid droplets and its gelation into irreversible fibrillar condensates.

### Cation-π Interactions Participate in FUS Phase Separation

Our observation that differential methylation of arginines in the C-terminal structured domain of FUS can modulate phase separation in a salt-dependent manner raises the question of what these arginines interact with during condensation. FUS contains only 5 acidic residues in the LC domain, making these unlikely to be the principal drivers of the implied electrostatic interaction. However, FUS has 27 tyrosines (but no tryptophan or phenylalanine) in the LC domain, which might allow protons in the guanidino moiety on the arginine side-chains to form cation-π interactions with electrons in the benzene ring of tyrosines.

To explore this idea, we investigated phase separation in purified full-length FUS proteins in which (1) multiple arginines in the sCTD were mutated to alanine or lysine or (2) multiple tyrosines in the LC domain were mutated to alanine or phenylalanine. In the arginine mutagenesis studies, we focused on the six arginines (216, 259, 407, 472, 473, and 476) that showed the greatest variability in methylation state after AdOx treatment. In the tyrosine mutagenesis studies, we focused on seven “near core” tyrosines (hereafter “ncYs”: 113, 122, 130, 136, 143, 149, and 161) adjacent to the β-sheet-forming core of the LC domain (amino acids [aa] 39–95). We chose not to investigate tyrosine replacement in the core LC domain to avoid confounding the experiment by disrupting the ability of the core domain to form anti-parallel β sheet assemblies.

**Figure S2:**
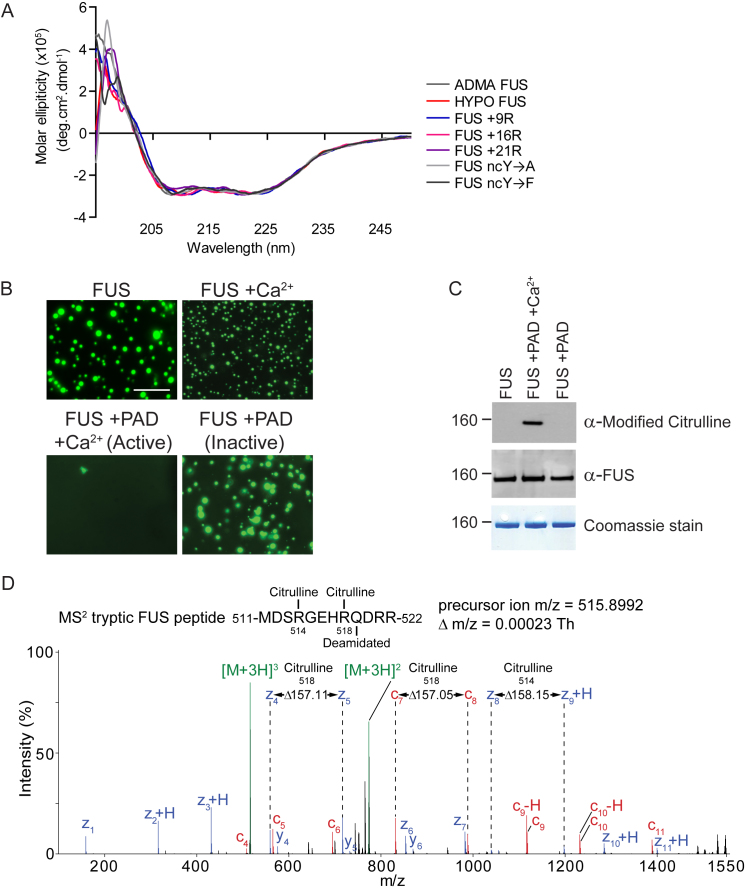
Hypomethylated FUS and FUS with Additional Arginines Have CD Spectra, Are Indistinguishable from ADMA Citrullination of FUS, and so Are Likely to Be Properly Folded; Protein Arginine Deiminase Treatment Converts Arginines to Citrulline, Related to Figure 2 (A) Circular dichroism (CD) spectrum of ADMA FUS, Hypo-FUS, 9R, 16R, 21R, ncY→A, ncY→F were measured on a JASCO-810 Spectropolarimeter at 25°C. 5μM of each purified protein was placed in a 1 mm path length quartz cuvette and the far-UV spectrum recorded in the wavelength range of 195 – 250 nm. Scans were repeated ten times and then averaged to yield a final spectrum for each construct. (B) At 1μM FUS and 50 mM NaCl, FUS undergoes phase transition. Upon addition of active PAD (in presence of calcium) phase transition is abrogated. (C) Representative western blots showing: *top panel:* anti-modified citrulline antibody detects a band in FUS + active PAD sample. *Middle panel:* equal FUS protein loading is detects by anti-FUS antibody. *Bottom panel:* Coomassie staining also detects equal FUS protein loading in each sample. (D) Orbitrap ETD MS^2^ spectrum of a tryptic FUS peptide citrullinated at R514 and R518 identified with confidence exceeding 99% from a PAD treated FUS preparation.

**Figure S3:**
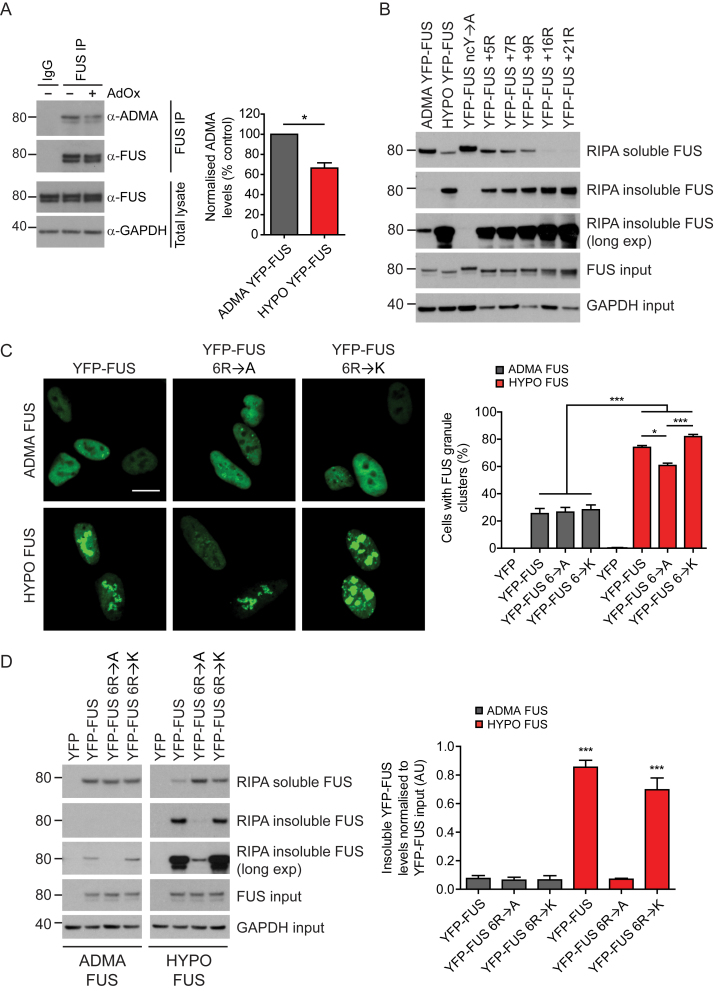
Substitution of Alanine for Six Arginines that Are Differentially Methylated Reduces FUS Aggregation Propensity after AdOx Treatment, whereas Lysine Substitution Still Supports FUS Aggregation Propensity, Related to Figure 3 (A) AdOx treatment causes hypomethylation of YFP-FUS in SH-SY5Y cells. Representative image of a western blot of immunoprecipitation of YFP-FUS from SH-SY5Y cells showing a significant reduction in asymmetrically dimethylated arginine (ADMA) epitopes after AdOx treatment. Unpaired t test, n = 5, ^∗^p < 0.05, error bars = SEM. (B) Western blot analysis of RIPA soluble and insoluble FUS in SH-SY5Y cells expressing YFP-FUS with or without AdOx treatment (HYPO and ADMA FUS respectively), or mutant FUS including FUS with fewer tyrosine residues (ncY→A) or more arginine residues (quantified in Figure 3E). Cells were lysed directly into loading buffer to determine FUS input. Hypomethylation of FUS results in increased levels of insoluble FUS with a concomitant decrease in soluble FUS, as does increasing the number of arginine residues, in a dose-dependent manner. ADMA and ncY→A FUS are predominately soluble but a longer exposure shows that FUS ncY→A is more soluble compared to ADMA FUS. (C) Representative confocal images of SH-SY5Y cells expressing YFP-FUS with 6 arginine residues mutated to alanine (6R→A) or lysine (6R→K), with DMSO (ADMA FUS) or AdOx treatment (HYPO FUS), with quantification of the number of cells with nuclear granule clusters on the right. Scale bar = 10 μm. Number of cells counted > 100. One-way ANOVA with Tukey posthoc test, n = 3, ^∗^p < 0.05, ^∗∗∗^p < 0.001, error = SEM. (D) Mutating the same 6 arginine residues to alanine (6R→A), but not lysine (6R→K), leads to reduced levels of AdOx induced (HYPO FUS) RIPA insoluble FUS compared to wild-type with quantification of the amounts of RIPA insoluble FUS on the right. One-way ANOVA with Tukey posthoc test, n = 4, ^∗∗∗^p < 0.001, error = SEM.

FUS phase separation was abrogated when cation-π interactions were disrupted by (1) replacement of arginines with alanine (FUS 6R→A) (Figure 2A), (2) enzymatic conversion of arginine to citrulline (Cit-FUS) by protein arginine deiminase (PAD), which replaces the positively charged ketimine group ( = NH) with an uncharged ketone group ( = O) (Figures 2A, 2E, and S2), and (3) conversion of the ncYs to alanine (FUS ncY→A) (Figures 2A and 2D). However, phase separation was maintained when cation-π interactions were preserved by (1) substitution of the arginines with lysine (FUS 6R→K), which has a cationic side chain, or (2) substitution of ncYs by phenylalanine (FUS ncY→F), which contains an aromatic ring in its side-chain (Figures 2A, 2C, and 2D).

Next, we investigated the effect of increasing the cation-π drive by strategic substitution of 9, 16 or 21 additional arginines to create more RGG and GRG motifs in the sCTD (FUS +9R, FUS +16R, and FUS +21R, respectively). The circular dichroism (CD) spectra of these proteins were indistinguishable from either ADMA FUS or HYPO FUS, implying that they were properly folded (Figure S2A). However, these constructs had significantly increased propensity to phase separate (as measured by turbidity; Figure 2B) and form gel-like structures (as measured by increasing numbers of nonspherical condensates; Figures 2C and 2D). This behavior was strongly dependent on the number of extra arginines (Figures 2A and 2B). To confirm that this effect was due to enhanced cation-π interactions, we mutated the ncYs to alanine in the construct with 16 extra arginines (FUS +16R ncY→A), thereby reducing the number of tyrosines available to form cation-π interactions with the extra arginines in the parental FUS +16R construct. This FUS +16R ncY→A construct rescued liquid droplet formation (Figures 2A, 2C, and 2D).

To examine the implied cooperativity between the tyrosine-rich disordered LC domain and the arginine-rich sCTD we separately purified these two domains and investigated their phase behavior alone or mixed together. The EmGFP-tagged LC fragment formed droplets and gels when cooled at high concentrations (>50 μM FUS), but did not phase separate at 1 μM at 23°C, even when mixed with dextran (Figure 2F). The mCherry-tagged arginine-rich sCTD also showed minimal phase separation under these conditions (Figure 2F). In contrast, robust phase separation rapidly occurred when the LC and sCTD fragments were mixed at 1:1 molar ratios, even in the absence of crowding agents (Figure 2F). However, unlike condensates from full-length FUS, these condensates were unstable, and dissolved within minutes, implying that full stabilization of condensed polymers requires tethering of the LC domain to the sCTD.

### Arginine:Tyrosine Cation-π Interactions Modulate FUS Phase Separation in Cells

Next, we investigated FUS phase separation in SH-SY5Y cells transiently expressing either YFP-tagged full-length FUS or YFP-tagged versions of the arginine or the tyrosine-modified FUS constructs described above. These cells were then treated for 24 hr with either DMSO control or varying doses (0–20 μM) of AdOx. Hypomethylation of FUS was confirmed by western blotting (Figure S3A).

**Figure 3:**
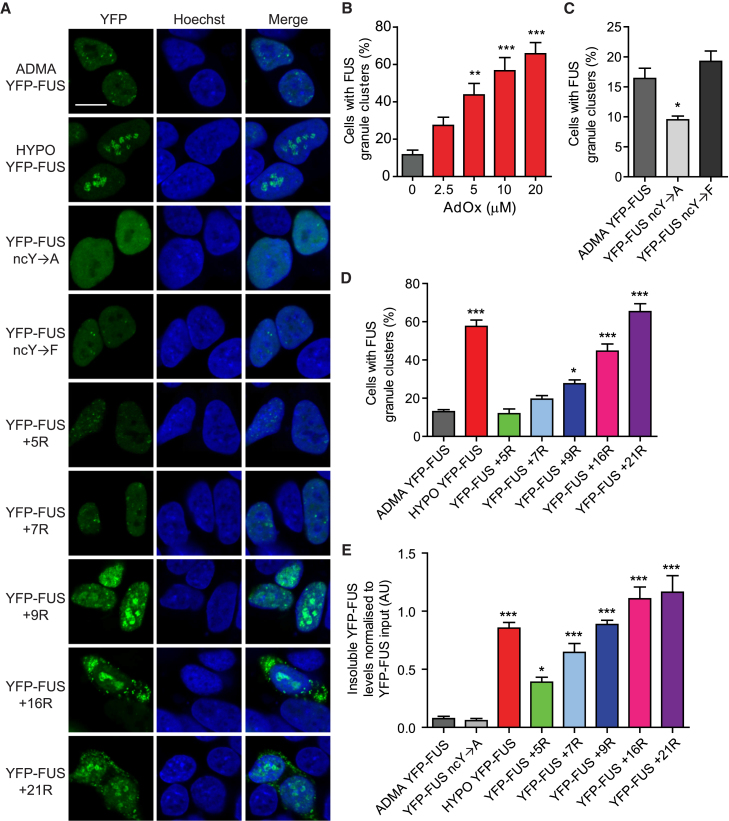
FUS Phase Separation in SH-SY5Y Cells Is Modulated by FUS Methylation, by the Number of Tyrosines near the LC Core (ncY), and Number of Arginines in the sCTD For a Figure360 author presentation of Figure 3, see the figure legend at https://doi.org/10.1016/j.cell.2018.03.056. (A) Representative images of FUS granules in SH-SY5Y cells expressing either YFP-tagged FUS, with or without AdOx treatment (HYPO or ADMA FUS respectively), or FUS with variations in the number of tyrosines or arginine. More cells had granules after AdOx treatment (HYPO FUS) and after expressing FUS with additional arginines (e.g., FUS +9R etc). Fewer cells had FUS granules after expressing FUS with tyrosines converted to alanine (ncY→A). Cells with tyrosines converted to phenylalanine (ncY→F) had normal granule formation. Scale bar, 10 μm. (B) AdOx causes a dose-dependent increase in cells with FUS granules. n = 100–200 cells/replicate experiment. Mean ± SEM, n = 5 replicates ^∗∗^p < 0.01, ^∗∗∗^p < 0.001, one-way ANOVA with Dunnett’s post hoc test. (C) Quantification of the number of cells in (A) with clusters of condensed FUS granules, comparing cells expressing wild-type FUS versus cells expressing FUS in which tyrosine residues in the N-terminal LC domain are mutated to alanine (inhibits cation-π interactions) or mutated to phenylalanine (maintains cation-π interactions). n > 200 cells/replicate. One-way ANOVA with Dunnett post hoc test, n = 3–7 independent replications, ^∗^p < 0.05, ^∗∗∗^p < 0.001. Error bars, SEM. (D) Quantification of the number of cells in (A) with clusters of condensed FUS granules, comparing cells expressing wild-type FUS versus cells expressing FUS with increasing numbers of additional arginine residues in the structured C-terminal domain. n > 200 cells/replicate. One-way ANOVA with Dunnett post hoc test, n = 3–7 independent replications, ^∗^p < 0.05, ^∗∗∗^p < 0.001. Error bars, SEM (E) Quantification of RIPA-insoluble FUS, normalized to input, in cells expressing ADMA FUS, HYPO FUS, FUS ncY→A, or FUS with additional arginines. One-way ANOVA with Dunnett’s post hoc test, n = 4, ^∗^p < 0.05, ^∗∗∗^p < 0.001. Error bars, SEM. See also Figure S3. Figure360: An Author Presentation of Figure 3

The results of these cellular studies were in good agreement with our initial biochemical studies. Thus, SH-SY5Y cells expressing FUS ncY→F, which have intact cation-π drive, were indistinguishable from cells expressing ADMA FUS (Figure 3C). Similarly, AdOx treatment and expression of FUS constructs with additional arginines caused a significant increase in the number of cells displaying intracellular FUS granules (Figures 3A and 3B; p < 0.01). The visible FUS aggregates were accompanied by increased abundance of FUS in RIPA-insoluble fractions of cell lysates (Figures 3A, 3D, 3E, and S3B). Crucially, the magnitude of these increases were dependent on the AdOx dose or number of extra arginines respectively. By contrast, FUS granule formation was significantly reduced in cells expressing FUS variants that diminish cation-π drive (either FUS ncY→A or FUS 6R→A) (Figures 3C and S3B–S3D).

These experiments support the notion that differential methylation of arginines in the sCTD of FUS can regulate FUS assembly through modification of cation-π interactions with tyrosines in the N-terminal LC domain.

### TNPO1, but Not EWS or TAF15, Acts as a Molecular Chaperone for FUS

**Figure 4:**
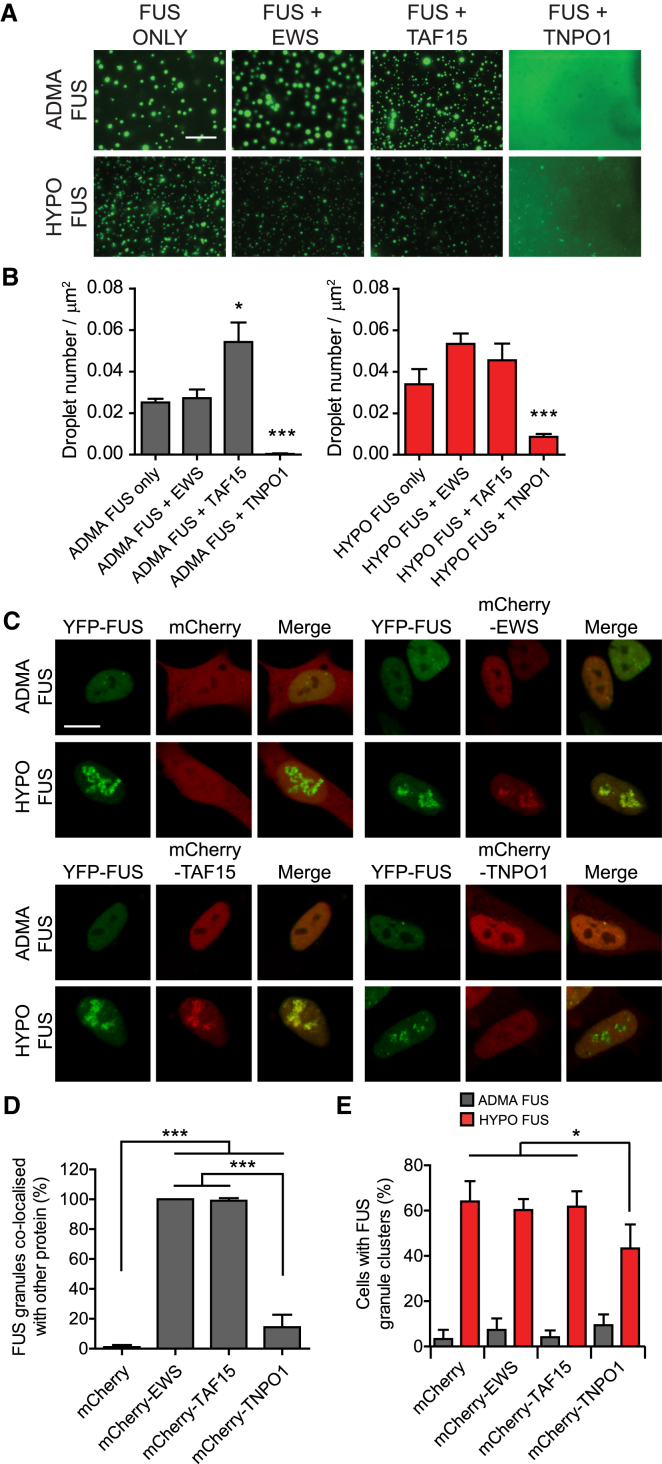
TNPO1 Is a Molecular Chaperone for ADMA FUS and HYPO FUS (A) Representative images of FUS phase separation in the presence of equimolar concentrations EWS, TAF15, or TNPO1. EWS and TAF15 had minimal impact on FUS phase separation. TNPO1 suppressed both ADMA FUS and HYPO FUS phase separation. Scale bar, 25 μm. (B) Quantification of (A). Kruskal-Wallis test, Dunn’s post hoc, n = 32–35 fields of view, ^∗^p < 0.05, ^∗∗∗^p < 0.001. Error bars, SEM. (C) Representative images of FUS granules in AdOx-treated SH-SY5Y cells expressing YFP-FUS (green) and mCherry, mCherry-tagged EWS, mCherry-tagged TAF15, or mCherry-tagged TNPO1 (red). FUS granules co-localized with EWS and TAF15, but not with TNPO1 or mCherry alone. Scale bar, 10 μm, n = 8 replicates. (D) Quantification of (C): FUS co-localized with EWS (100% ± 0.0%) and TAF15 (99.1% ± 1.7%). FUS poorly colocalized with TNPO1 (14.3% ± 8.5%) or mCherry only (1.0% ± 1.4%). One-way ANOVA, Tukey’s post hoc, n = 8 replicates, ^∗∗∗^p < 0.001. (E) TNPO1 reduced the number of SH-SY5Y cells with AdOx-induced FUS granules. One-way ANOVA, Tukey’s post hoc, n = 8 replicates, ^∗^p < 0.05. See also Figure S4.

Because hypomethylated FUS deposits in FTLD-FUS brain tissue contain EWS, TAF15 and TNPO1, we wondered whether these proteins might modulate FUS phase behavior. We therefore performed protein mixing experiments in which purified ADMA FUS or HYPO FUS were mixed with equimolar concentrations of TNPO1, EWS, TAF15, BSA, or buffer alone, and the phase separation behavior was investigated as above. When ADMA FUS or HYPO FUS were mixed with TAF15, EWS, or BSA, their phase behavior was essentially indistinguishable from ADMA FUS or HYPO FUS alone (Figures 4A and 4B). However, TNPO1 strongly suppressed phase separation of both ADMA FUS and HYPO FUS (Figures 4A, fourth column, and 4B, p < 0.001, n = 6 replications).

**Figure S4:**
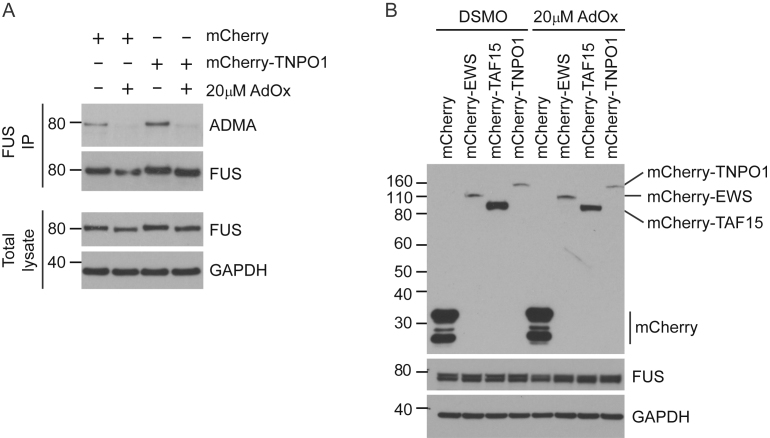
TNPO1 Expression Does Not Affect FUS Expression and Does Not Change Asymmetric Methylation Caused by AdOx Treatment, Related to Figure 4 (A) FUS immunoprecipitation and western blot studies show that overexpression of mCherry-TNPO1 has no effect on total YFP-FUS expression or FUS methylation. Representative of n = 3. (B) mCherry western blotting studies show that AdOx treatment has no effect on TNPO1, EWS, TAF15 or FUS expression. Representative of n = 3.

Similar results were obtained in cell-based experiments in SH-SY5Y cells expressing YFP-FUS together with mCherry vector alone, mCherry-EWS, mCherry-TAF15, or mCherry-TNPO1. Thus, although both EWS and TAF15 colocalized with HYPO FUS granules induced by AdOx, their co-expression had no impact on granule formation (Figure 4C, top-right and bottom-left panels; Figure 4D, p < 0.001, n = 8 replications). By contrast, TNPO1, suppressed FUS granule formation, and TNPO1 was largely absent from HYPO FUS granules that did form (Figure 4C, lower-right panel; Figure 4D; p < 0.001, Figure 4E, p < 0.05, n = 8 replications). These inhibitory effects of TNPO1 were not attributable to (1) TNPO1-induced changes in the abundance or methylation state of FUS (Figure S4A) or (2) AdOx-induced changes in the abundance of EWS, TAF15 or TNPO1 (Figure S4B).

Taken together, these experiments lead to the intriguing conclusion that TNPO1 may act as a cellular molecular chaperone for both ADMA FUS and HYPO FUS.

### Biophysical Analysis of FUS Phase Separation Probed with Amyloidophylic Dyes

To gain insight into the secondary and quaternary structures of ADMA FUS and HYPO FUS assemblies during phase separation, we applied two complementary approaches. The first approach employed the amyloidophylic fluorescent dyes—thioflavin T (ThT) and pentameric formyl thiophene acetic acid (pFTAA)—as chemical probes that could be applied to both protein and cellular preparations. The second approach employed atomic force microscopy-basedinfrared nanospectroscopy (AFM-IR; see the next section).

**Figure S5:**
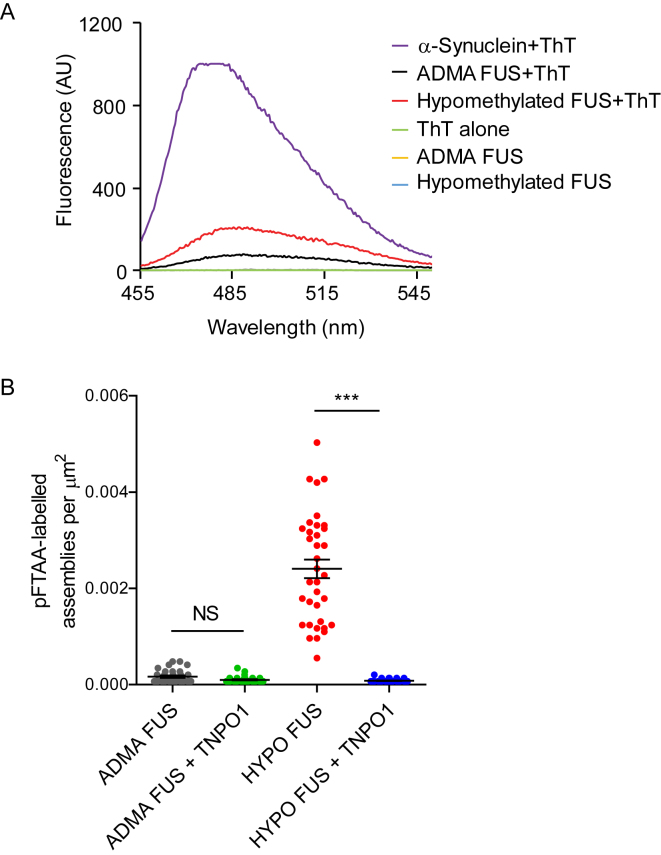
pFTAA Binds and Fluoresces with HYPO FUS but Not ADMA FUS, whereas ThT Only Binds and Fluoresces Very Weakly to HYPO FUS or ADMA FUS, Related to Figure 5 (A) Phase-separated FUS assemblies only weakly bind ThT. Fluorescence spectroscopy reveals weak ThT binding to methylated FUS (black line) but stronger ThT binding to hypomethylated FUS (red line). No fluorescence was detected from methylated FUS (yellow line), hypomethylated FUS (blue line) or ThT alone (green line). ThT binding to α-synuclein generated > 5-fold greater fluorescence (purple line). (B) Phase-separated FUS assemblies bind pFTAA, especially hypomethylated FUS. Plot of pFTAA fluorescent assemblies perμm^2^ for either ADMA FUS or hypomethylated FUS with and without TNPO1. There is strong pFTAA fluorescence from hypomethylated FUS assemblies which is dramatically reduced in the presence of equimolar amounts of TNPO1. Kruskal-Wallis with Dunn’s post hoc, n > 40 droplets over 3 replications, ^∗∗∗^p < 0.001, NS = not significant, error bars = SEM.

ThT showed only minimal binding and fluorescence enhancement upon addition to either purified ADMA FUS protein condensates (Figure S5A, black line) or purified HYPO FUS protein condensates (Figure S5A, red line). By comparison, equimolar concentrations of α-synuclein (a conventional amyloid-forming protein) displayed robust ThT binding and fluorescence (Figure S5A, purple line). In cell-based experiments, ThT displayed minimal binding and fluorescence to HYPO FUS in AdOx-treated SH-SY5Y cells (data not shown). This result is in good agreement with prior studies showing poor binding of ThT to pathological fibrillar FUS assemblies in human FTLD-FUS and fALS-FUS tissues and in *C. elegans* models (Urwin et al., 2010). Further work with ThT was abandoned. However, pFTAA showed more promising results.

**Figure 5:**
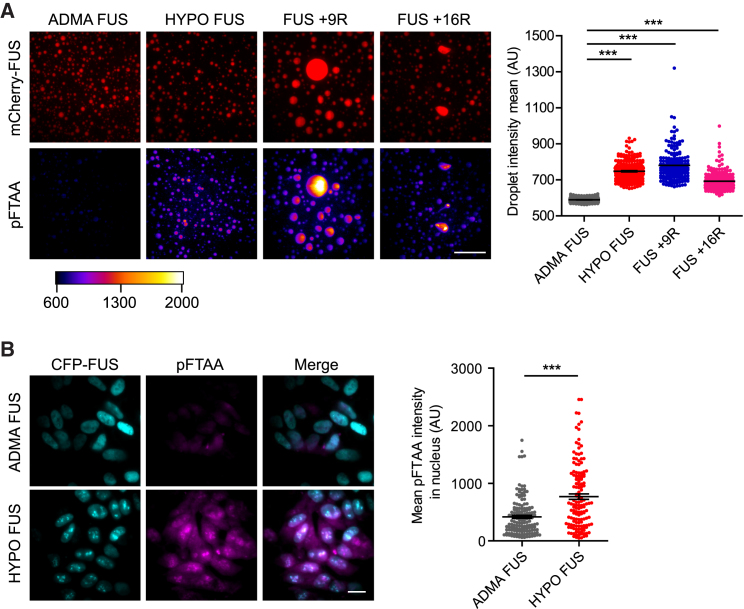
pFTAA Differentially Binds and Fluoresces with FUS Hydrogel Condensates (A) Representative images of mCherry-tagged FUS (red, top row) labeled with pFTAA (bottom row). ADMA FUS weakly binds pFTAA. AdOx-treated (HYPO FUS) FUS +9R and FUS +16R strongly bind pFTAA. Kruskal-Wallis test, Dunn’s post hoc, n > 190 droplets, n = 3 independent replications, ^∗∗∗^p < 0.001. Error bars, SEM. Scale bar, 20 μm. (B) AdOx-treated cells have intracellular HYPO FUS granules that co-stain with pFTAA (bottom row). Mann-Whitney U test, n > 140 cells over six fields of view, ^∗∗∗^p < 0.0001. Error bars, SEM. Scale bar, 20 μm. See also Figure S5.

pFTAA is a high-affinity, cell permeant, luminescent oligothiophene dye that discriminates different conformers of β-sheet-containing aggregates of tau and PrP (Klingstedt et al., 2013). pFTAA showed modest binding and fluorescence with ADMA FUS condensates (Figure 5A, first column of images), but significantly greater binding and fluorescence with condensates composed of HYPO FUS, FUS +9R, or FUS +16R (Figure 5A, p < 0.001). Crucially, pFTAA was also able to detect the chaperone-like activity of TNPO1. Thus, premixing TNPO1 with ADMA FUS or HYPO FUS dramatically reduced the number of assemblies that bound pFTAA (Figure S5B, p < 0.001). In cell-based experiments, pFTAA displayed significant binding and fluorescence with HYPO FUS condensates in AdOx-treated SH-SY5Y cells (Figure 5B).

These experiments suggest that liquid droplet condensates of ADMA FUS contain a small proportion of FUS in an antiparallel β sheet conformation, and this antiparallel β sheet content is then significantly increased upon conversion to hydrogel-like HYPO FUS condensates.

### Structural Analysis of FUS Phase Separation by AFM-IR Nanospectroscopy

To further explore the relationship between the three-dimensional morphology and the secondary and quaternary structures of individual ADMA FUS and HYPO FUS assemblies, we next applied a recently developed single-molecule technique that combines atomic force microscopy (AFM) with infrared nanospectroscopy (IR) (Dazzi et al., 2012). In contrast to conventional bulk approaches, AFM-IR provides a unique tool to probe, at nanoscale resolution, the morphological, nanomechanical, chemical, and secondary and quaternary structural properties of individual protein assemblies, a feature crucial for characterizing heterogeneous molecular systems (Ruggeri et al., 2015b).

**Figure 6:**
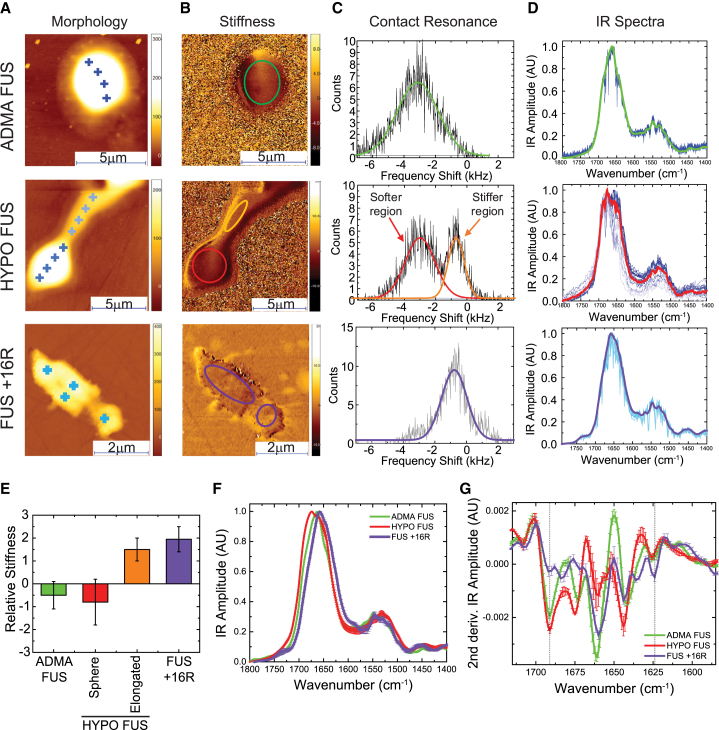
Nanoscale Resolution Analysis of the Mechanical and Secondary and Quaternary Structural Properties of Individual FUS Condensates Reveal Substantial Differences between ADMA FUS versus HYPO FUS and Cation-π-Enhanced FUS Condensates For a Figure360 author presentation of Figure 6, see the figure legend at https://doi.org/10.1016/j.cell.2018.03.056. (A) Representative AFM 3D morphology maps of individual ADMA FUS (top), HYPO FUS (center), and FUS +16R granules (bottom). Crosses represent position of nanoscale localized IR spectroscopy measurements. (B) Representative tip-sample contact resonance maps of nanoscale stiffness for ADMA FUS (top), HYPO FUS (middle), and cation-π enhanced FUS +16R condensates (bottom). Colored circles indicate where contact resonance shift was evaluated. (C) Histogram of tip-sample contact resonance shift (“stiffness”) for representative ADMA FUS (top), HYPO FUS (middle), and FUS +16R (bottom) condensates. ADMA FUS and FUS +16R condensates have homogeneous (but different) nanomechanical properties. HYPO FUS condensates are heterogeneous, with softer and stiffer regions. The colored average curves correspond to the distribution of contact resonance shifts in the colored regions in (B). (D) Individual nanoscale raw localized spectra and their average (bold) from locations indicated by “+” on AFM maps for corresponding ADMA FUS (top), HYPO FUS (center), and FUS+16R (bottom) condensates. n = 3 independent methylated ADMA FUS assemblies; n = 4 independent hypomethylated FUS assemblies; n = 4 for the FUS +16R assemblies. Error bars, SEM. (E) Relative stiffness of the FUS assemblies. ADMA FUS (green) and round HYPO FUS (red) display soft nanomechanical properties. The non-spherical HYPO FUS and FUS+16R, display stiffer properties. n ≥ 3 independent ADMA FUS; HYPO FUS; FUS +16R condensates. Error bars, SD. (F) Average IR spectra in amide band I and II for ADMA, HYPO, and FUS +16R, which derive from the average of the average of 55 ADMA FUS, 73 HYPO FUS, and 88 FUS +16R spectra. Error bars, SEM. (G) Deconvolution of amide band I reveals that (1) HYPO FUS droplets (red line) have a significant increase of antiparallel β sheet, random coil and β-turn structures, compared to the ADMA FUS droplets (green line). (2) FUS +16R assemblies (purple line) are stabilized by parallel amyloidogenic β sheet content (1,625 cm^−1^). Error bars, SEM. See also Figure S6. Figure360: An Author Presentation of Figure 6

We applied this approach to investigate ADMA FUS, HYPO FUS and FUS +16R condensates generated using the same conditions used in earlier experiments. The condensates were placed on zinc selenide (ZnSe) windows. AFM-IR was then used to acquire nanoscale resolved maps on the 3D morphological (Figure 6A) and nanomechanical properties of the assemblies (Figure 6B). The nanomechanical state was assessed by measuring the shifts in the tip-sample contact resonance (Figure 6C), which varies monotonically as a function of the intrinsic Young’s modulus of the sample (Dazzi et al., 2012). Because of the complexity of defining the absolute nanomechanical properties of soft biological samples, we measured the stiffness of each assembly relative to the stiffness of the underlying ZnSe window. We then acquired nanoscale resolved infrared (IR) spectra from several locations within each assembly (denoted by “+” in Figure 6A). Because each nanospectroscopy measurement has a lateral resolution down to 20 nm (Ruggeri et al., 2015b), it allows exquisite characterization of the chemical properties and secondary and the quaternary chemical structures across multiple locations in individual condensates (Galante et al., 2016). The average spectrum from all assemblies for each group was then calculated (Figure 6F), and the corresponding average second derivatives in amide band I (Figure 6G) were evaluated to extract the principal structural components of the condensates. Cumulatively, we acquired 216 spectra with corresponding detailed nanomechanical data from ADMA FUS, HYPO FUS, and FUS +16R assemblies.

#### ADMA FUS: Homogeneous Spherical Liquid-like Structures with Low β Sheet Content

ADMA FUS condensates had relatively homogeneous morphological and mechanical properties, with high sphericity and low relative intrinsic stiffness akin to that of a liquid. In agreement with the nanomechanical data, the chemical responses of different ADMA FUS liquid droplets were also relatively homogeneous, all being composed of α-helical, native β sheet, random coil, β-turn, and residual antiparallel intermolecular/cross-β sheet structures (Figures 6D, top panel, and S6A–S6D).

#### HYPO FUS: Heterogeneous Assemblies with Liquid- and Gel-like Condensates

By contrast, HYPO FUS condensates were both morphologically and mechanically heterogeneous. Crucially, this heterogeneity existed both within individual HYPO FUS assemblies and between different HYPO FUS condensates (Figures 6A–6D, second row, and S6A). This *intra*-sample heterogeneity is quantitatively demonstrated in Figure 6 for a representative HYPO FUS condensate. This particle has a spherical component (red circle, middle panel, Figure 6B) fused to a non-spherical component (orange ellipse, middle panel, Figure 6B). The spherical component showed softer mechanical properties, like those of ADMA FUS condensates. The nonspherical component showed stiffer nanomechanical features more suggestive of a gel. This regional heterogeneity within a single condensate is of note because it suggests conversion between liquid droplet and hydrogel conformations.

**Figure S6:**
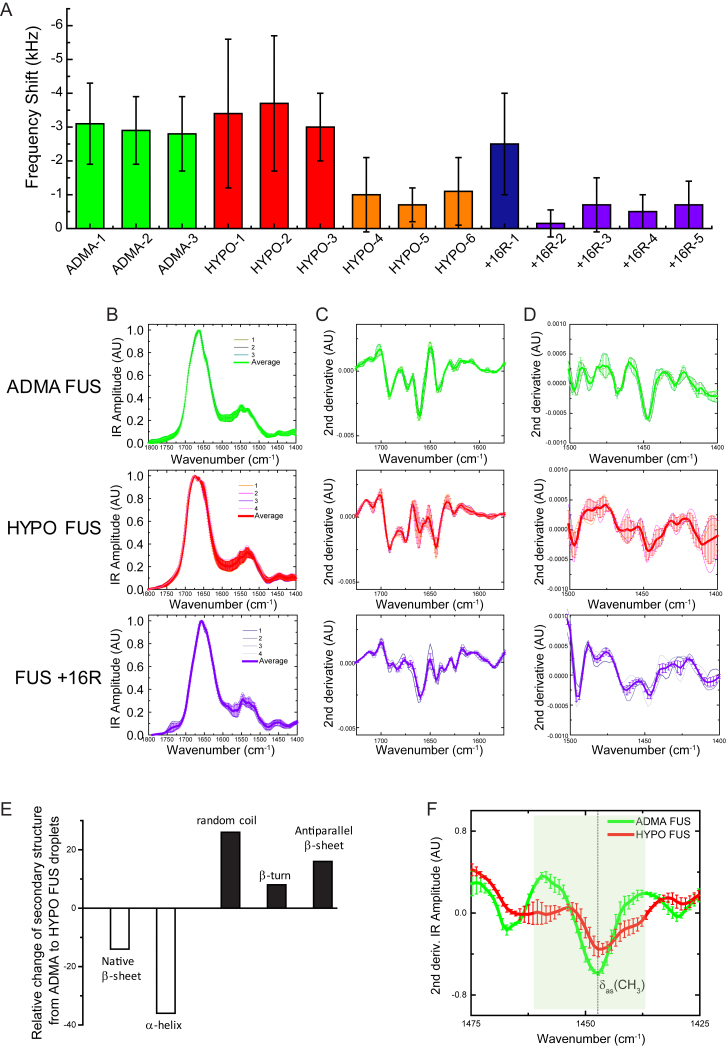
Nanoscale Infrared Spectroscopy Reveals that ADMA, HYPO FUS, and FUS+16R Assemblies Display Different Stiffness and Secondary and Quaternary Organization, Related to Figure 6 (A) AFM tip-sample Contact Frequency measurements of ADMA and HYPO FUS assemblies. The average frequency shift for 3 different ADMA-FUS droplets (green), for 6 regions within 4 different HYPO FUS droplets (red and orange) and 5 regions within 4 FUS +16R droplets, with the relative standard deviation. (B) Average IR spectrum of each measured ADMA, HYPO and FUS +16R granules and their average. (C and D) Second derivatives of IR spectra at specific wavenumbers of each individual ADMA, HYPO and FUS+16R granules and their average. We acquired a total of 55, 73, 88 spectra for the WT, HYPO and +16R droplets, respectively. ADMA and +16R droplets show higher degrees of homogeneity than HYPO ones, which show higher structural heterogeneity. (E) The relative conformational change between ADMA and HYPO -FUS assemblies is displayed as histograms, and shows increased random coil, β-turn and antiparallel β sheet content (black bars), and decreased native β sheet and α-helical content (white bars) in HYPO FUS assemblies. (D) HYPO FUS assemblies show lower and shifted signals of methyl group absorption (δ_as_(CH_3_), methyl asymmetric stretching), confirming a lower methylation state of HYPO FUS assemblies. Error bars = SEM.

IR spectra of HYPO FUS assemblies were also highly heterogeneous, and this heterogeneity correlated with the nanomechanical heterogeneity. The regions within a single droplet possessing higher stiffness were also the ones possessing higher absorption at 1,695 cm^−1^, corresponding to antiparallel cross-β sheets. More importantly, on average, deconvolution of amide band I of the HYPO FUS IR spectra revealed increased antiparallel cross-β sheet, random coil and β-turn content compared to ADMA FUS assemblies (Figures 6G, S6B, S6C, and S6E). Furthermore, amide band II of HYPO FUS assemblies was shifted toward lower wave-numbers, confirming independently increased hydrogen bonding (Figures 6G, S6B, S6C, and S6E). In addition, on average, HYPO FUS assemblies had reduced and shifted signals originating from the methyl group absorption (δ_as_(CH_3_), methyl asymmetric stretching at 1,445 cm^−1^), confirming their lower methylation state (Figure S6F).

Remarkably, at the level of individual HYPO FUS assemblies there was considerable heterogeneity in the spectral data. Thus, HYPO FUS condensates with liquid-like nanomechanical properties (e.g., within the red circle in Figure 6B) had IR spectra like those of ADMA FUS condensates. But the HYPO FUS condensates with stiffer nanomechanical properties (e.g., within the orange ellipse in Figure 6B) had higher antiparallel cross-β sheet content and increased intermolecular hydrogen bonding, suggesting the presence of a hydrogel-like structure (Ruggeri et al., 2015a).

#### FUS +16R: Stiff, Non-spherical Parallel β-Sheet-Rich Hydrogen-Bonded Assemblies

The FUS +16R condensates displayed a predominantly non-spherical 3D morphology and stiffer nanomechanical properties, like those of the gelled HYPO FUS condensates (Figures 6A–6D, lower panels). Quantitative analysis of the IR spectra for FUS +16R assemblies was complicated by partial overlap in the absorption spectra of the extra arginine side-chains, which absorb at 1,635–1,675 cm^−1^. As a result, it was not possible to quantitatively compare the secondary and quaternary structural composition of FUS +16R with HYPO FUS or ADMA FUS assemblies. Nevertheless, the spectra for FUS +16R reveal significant intermolecular amyloidogenic antiparallel (1,695 cm^−1^) and parallel β sheet (1,625 cm^−1^) content, related to the dense network of intermolecular hydrogen bonding (Ruggeri et al., 2015b), and lead to the observed stiffer nanomechanical properties of FUS +16R condensates.

These results support the notions that (1) both liquid droplet and hydrogel phase transitions are associated with increasing *inter*-molecular hydrogen bonding and increasing antiparallel cross-β sheet, random coil and β-turn structures and (2) these structural shifts are associated with selective binding of amyloidophyllic dyes, such as pFTAA. Finally, the AFM-IR analyses of FUS +16R condensates demonstrate that enhancing the arginine: tyrosine cation-π interaction by increasing the number of arginines in the C-terminal regions of FUS outside the core LC domain, promotes formation of stabilized hydrogels. This observation suggests that FUS constructs with additional arginines (“cation-π enhanced” constructs) can at least partially replicate the propensity of HYPO FUS to form pathologically stable FUS granules, and might therefore be useful as a molecular model of FTLD-FUS (see below).

### Arginine Methylation Status Regulates Neuronal FUS RNP Granule Function

The experiments described above support our hypothesis that FUS phase transition can be regulated by (1) methylation of arginines in the sCTD of FUS and (2) interactions with TNPO1. We were therefore curious to determine whether manipulation of the arginine methylation status of FUS and its interaction with TNPO1 might alter FUS RNP granule function in distal neuron terminals.

**Figure 7:**
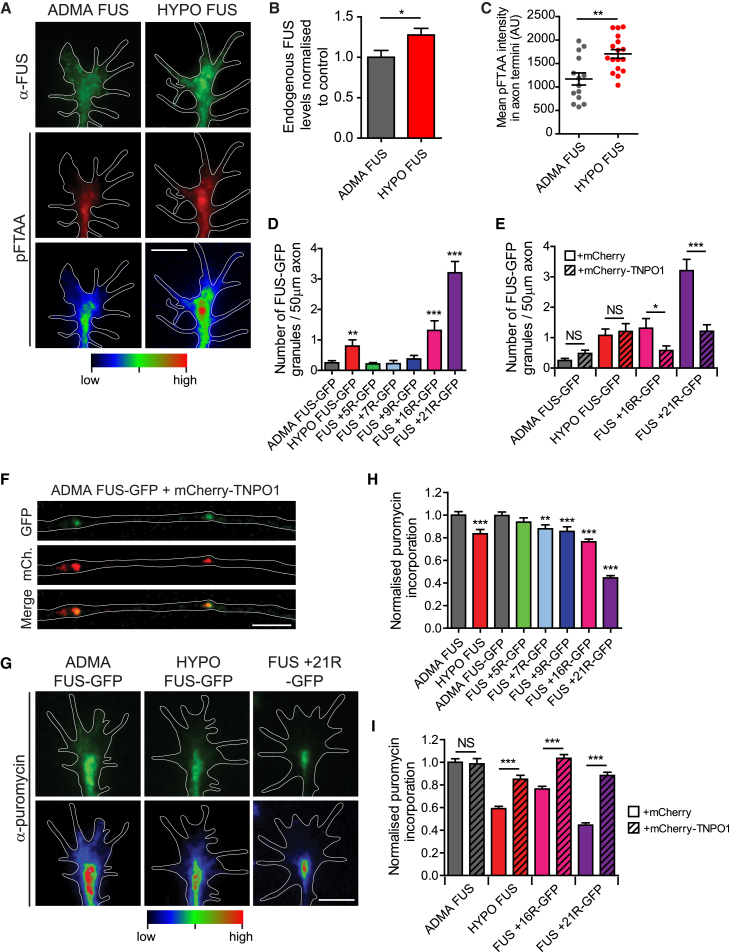
Hypomethylation of FUS or FUS Constructs with Additional Arginines Promote FUS Granule Formation and Attenuate Axonal New Protein Synthesis, which Is Rescued by TNPO1 For a Figure360 author presentation of Figure 7, see the figure legend at https://doi.org/10.1016/j.cell.2018.03.056. (A) Representative images of endogenous FUS (anti-FUS antibody, green) and pFTAA binding (red and heatmaps) in fixed axon terminals, showing AdOx-induced increased FUS aggregates and pFTAA binding (right). Scale bar, 5 μm. (B) Quantification of the increased accumulation of endogenous FUS granules following AdOx treatment of the axon terminals in (A). The accumulation of FUS granules was assessed by FUS immunofluorescence. Unpaired t test, n = 26 and 16 axon terminals. ^∗^p < 0.05, ^∗∗^p < 0.01. Error bars, SEM. (C) Quantification of the change in phase state of endogenous FUS granules following AdOx treatment of the axon terminals in (A). Phase state was assessed using pFTAA fluorescence intensity. Unpaired t test, n = 26 and 16 axon terminals, ^∗^p < 0.05, ^∗∗^p < 0.01. (D) AdOx treatment (HYPO FUS) or expression of FUS with additional arginines increases FUS granules in live distal axon segments. Unpaired t test, n = 20–30 axon segments, ^∗∗^p < 0.01, ^∗∗∗^p < 0.001. Error bars, SEM. (E) Number of FUS-GFP granules per 50 μm live distal axon segments following mock or AdOx treatment, or expressing FUS with additional arginines, and co-expressing mCherry or mCherry-TNPO1. Unpaired t test, n = 20–30 axon segments, ns, not significant, ^∗^p < 0.05, ^∗∗∗^p < 0.001. Error bars, SEM. Video S3 displays movement of TNPO1 (red) in FUS granules in the axon shaft of neurons expressing ADMA FUS. (F) Representative images showing colocalization of FUS and TNPO1 in distal axons. Scale bar, 5 μm. (G) Representative images (pseudo-colored green) and heatmaps of puromycin-labeled newly synthesized proteins in mock-treated (ADMA FUS, left), AdOx-treated (HYPO FUS, middle), or FUS +21R-GFP-expressing axon terminals (FUS +21R, right). Scale bar, 5 μm. (H) Quantification of (G). Unpaired t test, n > 100 axon terminals. ns, not significant. ^∗∗^p < 0.01, ^∗∗∗^p < 0.001. Error bars, SEM. (I) Coexpression of mCherry-TNPO1 rescues new protein synthesis in AdOx-treated neurons and neurons expressing FUS with additional arginines. Unpaired t test, n > 100 axon terminals, ns, not significant, ^∗∗∗^p < 0.001. Error bars, SEM. See also Video S3. Figure360: An Author Presentation of Figure 7

To address this question, we examined FUS assembly, FUS conformational state, and FUS RNP granule function in *ex vivo Xenopus* retinal neuron cultures prepared as previously described (Lin and Holt, 2007). The distribution of FUS assemblies was assessed in mock-treated or AdOx-treated axons (20 μM for 30 min) either by anti-FUS immunofluorescence (for endogenous FUS) or by GFP fluorescence (for live imaging of axonal FUS granules in neurons expressing GFP-tagged FUS) (Figures 7A–7F). A caveat to the use of AdOx to induce hypomethylation of FUS is the potential for AdOx to alter the methylation state of numerous other neuronal proteins. To circumvent this caveat, in parallel experiments, we also expressed the “cation-π enhanced” constructs in axon terminals (FUS +5R, FUS +7R, FUS +9R, FUS +16R, and FUS +21R).

In good agreement with our earlier biochemical and cellular experiments, AdOx treatment of isolated axons and axon terminals induced the formation of bright FUS granules that showed increased pFTAA fluorescence (Figures 7A–7F; p < 0.05, p < 0.01), Crucially, the parallel experiments in neurons expressing “cation-π enhanced” constructs revealed an arginine dose-dependent increase in similar axonal FUS granules (Figure 7D, p < 0.001). pFTAA labeling was not investigated in neurons expressing “cation-π enhanced” constructs because the excitation/emission spectra of their GFP tags overlap those of pFTAA.

### Hypomethylated FUS Assemblies Impair Neuronal New Protein Synthesis

Next, we used puromycin labeling of nascent proteins in isolated axon terminals (Lin and Holt, 2007) to assess the impact of AdOx (HYPO FUS) and of “cation-π enhanced” constructs on FUS RNP granule function (Figures 7G–7I). These experiments demonstrated that new protein synthesis was significantly attenuated in both AdOx-treated axon terminals (HYPO FUS), and in the axon terminals expressing “cation-π enhanced” constructs (Figures 7G–7I; p < 0.001). Crucially, the magnitude of this effect (∼0.60–0.80 of control) approximated that of fALS-FUS mutations (∼0.80 of control) (Murakami et al., 2015).

### TNPO1 Rescues Impaired Protein Synthesis in Axon Terminals

Outside of the nucleus, TNPO1 colocalizes with some cytoplasmic RNA granules, where it coexists with FUS, purine-rich element binding protein A (Pur-α, which modulates toxicity of ALS-associated FUS mutants), and Staufen-1 (a marker of neuronal transport granules) (Jain et al., 2016). In this non-nuclear role, TNPO1 facilitates the import of protein components into RNA granules in a Ras-related nuclear protein-GTP-independent fashion (Twyffels et al., 2014). In agreement with this prior work, we found that mCherry-TNPO1 is also present within motile granules in axon terminals (Video S3).

Given that TNPO1 is expressed in axon terminals, and may function as a molecular chaperone, we next investigated whether modest overexpression of TNPO1 might restore FUS RNP granule function in axon terminals treated with AdOx or expressing “cation-π enhanced” FUS constructs. As predicted, mCherry-TNPO1 fully rescued new protein synthesis in both AdOx-treated neurons and in neurons expressing “cation-π enhanced” constructs (Figures 7E and 7I, p < 0.001). However, mCherry-TNPO1 had no effect on new protein synthesis in mock-treated (ADMA FUS) axon terminals.

## Discussion

The experimental data described here reveal that the phase behavior of FUS is modulated by (1) the post-translational methylation state of arginines in the structured C-terminal domain and (2) by molecular chaperones such as TNPO1. This conclusion provokes several broad lines of thought.

### Cation-π Interactions and Cooperativity between N- and C-Terminal Domains

Previous reports have shown that the N-terminal LC domain of FUS (residues 1–214) is necessary and sufficient for phase separation and gelation of FUS, and does so by forming intermolecular β-sheet-rich fibrils. The experiments described here support and extend this view. Thus, our AFM-IR experiments clearly reveal that intermolecular hydrogen bonding between β sheet regions contributes to both liquid droplet and hydrogel formation.

However, our data also lead to the conclusion that FUS phase separation is regulated by additional factors beyond just the intermolecular β-sheet-forming LC domain. The experiments described here reveal that phase separation is also driven by multivalent cation-π interactions, which occur physiologically between multiple arginines in the sCTD and multiple tyrosines in the LC domain. In support of this, we have shown that cation-π pairing and FUS phase separation are impeded by (1) replacement of C-terminal domain arginines by alanines, (2) conversion of these arginines to citrullines, or (3) replacement of N-terminal tyrosines with alanines. Conversely, we have shown that phase separation is maintained by amino acid replacements that preserve the cation-aromatic ring pairing (i.e., arginine to lysine; tyrosine to phenylalanine), rather than planar-π or planar-planar interactions. Finally, we have shown that phase separation is augmented in an arginine dose-dependent manner by increasing the number of arginines in the C-terminal domain, presumably by enhancing the number of cation-π interactions. Two further observations underscore the importance of domains outside the LC domain in promoting liquid phase separation and gelation of FUS. First, as shown here, FUS phase separation is also modulated by TNPO1, which binds to FUS via its structured C-terminal domain. Second, fALS-FUS mutations map predominantly to the C-terminal domain.

Additional work will be required to fully understand the mechanics of the co-operative interaction between the C-terminal domain and the intermolecular β-sheet-forming LC domain. Our mixing experiments suggest a model in which multivalent cation-π interactions initiate phase separation, thereby bringing LC domains close together in restricted volumes and at higher local concentration. We propose that this close apposition permits the formation of more stable, intermolecular hydrogen-bonded β-sheet-rich condensates driven by the LC domain. The transient nature of the condensation events that occur when physically separate LC domain and C-terminal domain proteins are mixed could arise through several mechanisms. For instance, the untethered C-terminal proteins might be less efficient in restraining the egress of LC domain peptides from nascent condensates during initial sol→droplet phase separation. Alternatively, the untethered arginine-rich C-terminal domain proteins might bind to tyrosines within the intermolecular β-sheet-forming parts of the LC domain, and interfere with further condensation.

### Converting Liquid Droplets to Gels

Our observation that some HYPO FUS assemblies contain regions that are liquid droplet-like while other regions are gel-like, suggests liquid droplet and hydrogel states are alternate but mechanistically related, and potentially inter-convertible states within a single FUS assembly. This interpretation is supported by theoretical work indicating that in systems close to a critical point, variations in interaction strength or solvation volume can allow coexistence of phase separation and gelation (Harmon et al., 2017).

### TNPO1 as a Chaperone in Non-nuclear Compartments

There are likely to be active processes to maintain FUS and other phase-separating proteins in a dispersed state, and to reverse gelled forms. As is the case for TDP43 (Götzl et al., 2016), autophagy and proteasome pathways are likely important components of this quality control system. However, molecular chaperones are also likely to be involved. Indeed, our experiments show that TNPO1 is a molecular chaperone for ADMA FUS and HYPO FUS that acts in distal axonal compartments of neurons, as well as at the nuclear pore.

### Methylation as a Physiological and Pathological Regulator

Our observations that arginine methylation status profoundly influences FUS phase separation, and that adding very small amounts of unmethylated FUS (≤5%) to ADMA FUS results in rapid phase separation and gelation raises the possibility that arginine methylation state might be a physiological method to regulate FUS phase behavior. This conclusion raises several critical questions. Which differentially methylated arginines are essential for a change in FUS phase behavior? How do unmethylated arginines increase the cation-π interaction strength? Are specific tyrosines more important than others? In some Tudor domain proteins, protein:protein binding occurs because of interactions between asymmetrically dimethylated arginines on one protein and specific clusters of aromatic amino acids on the other protein that are arranged into three-dimensional “aromatic cages” (Tripsianes et al., 2011, Zhang et al., 2017). Does a similar process occur during formation of the cation-π interactions in FUS? If so, do unmethylated arginines alter the thermodynamics of interactions with such “cages”? Or do these unmethylated arginines simply form promiscuous interactions with tyrosines that are not in specific clusters or cages, as proposed for other Tudor domain proteins (Zhang et al., 2017)? Further work will be required to address these questions. However, because the tyrosine-rich LC domain of FUS is natively disordered, if they exist, such tyrosine clusters or cages are unlikely to have the same highly ordered three dimensional structure of the tyrosine-based “aromatic cages” in Tudor domain proteins.

Finally, how is methylation and demethylation of individual arginines regulated, both physiologically and pathologically? Multiple PRMT enzymes are known, at least some of which are components of RNP granules (Scaramuzzino et al., 2013). However, to date, only a single arginine demethylase has been identified (Jumonji domain-containing 6-JMJD6) (Blanc and Richard, 2017).

### FUS and FTLD

While not directed at generating a model of FTLD-FUS, the experiments reported here have obvious implications for understanding this disorder. Our observation that HYPO FUS and fALS mutant FUS assemblies have similar biochemical and biophysical properties, and similar effects on FUS RNP granule function, suggests that gel-like assemblies of hypomethylated wild-type FUS and of fALS mutant FUS represent a common final mechanism for FUS-related neurodegeneration.

We speculate that neuron subtype-specific differences in molecular chaperones, methylases, and demethylases might account for why hypomethylated non-mutant FUS accumulates in fronto-temporal neurons in FTLD-FUS, while normally methylated fALS- mutant FUS accumulates in corticospinal and spinal neurons in fALS-FUS. Why FUS becomes hypomethylated and inadequately chaperoned in FTLD-FUS is unknown. Mutations have not been detected in the PRMT genes tested to date. However, our results offer other candidate genes that are worth investigating for disease-causing mutations, including TNPO1, PADs, and JMJD6.

One important difference between our model and FTLD concerns the fact that nuclear assemblies of FUS in FTLD contain EWS, TAF15, and TNPO1. In our model TNPO1 is largely absent from FUS aggregates. The explanation for this difference is not immediately apparent. It might arise from the fact that in our model the level of TNPO1 expression is not a limiting factor because it is overexpressed. In contrast, in neurons TNPO1 might be titrated out by an excess of hypomethylated FUS. Regardless, the work reported here provides a starting point to investigate how pathological methylation and how pathological phase separation of FUS that escapes from its normal molecular chaperones might be targeted therapeutically.

Clearly, several reagents and methods developed here including pFTAA, AFM-IR, and the cation-π-enhanced constructs will be useful tools to delve further into the biophysics of FUS phase separation, and to create molecular models of increased FUS phase separation propensity in FTLD-FUS.

## STAR★Methods

### Key Resources Table

**Table:** 

REAGENT or RESOURCE	SOURCE	IDENTIFIER
**Antibodies**		

Anti-FUS	Santa Cruz Biotechnology, Heidelberg, Germany	sc47711 RRID:AB_2105208
Anti-dimethyl-arginine asymmetric	Merck Millipore, Watford, UK	ASYM24 RRID:AB_310596
Anti-GAPDH	Cell Signaling Technology, Danvers, USA	5174S RRID:AB_10622025
Anti-Puromycin-AlexaFluor647	Merck Millipore, Watford, UK	MABE343-AF647
Rabbit polyclonal anti-mCherry	Abcam	Cat# ab167453; RRID:AB_2571870
Anti-Modified Citrulline Antibody, clone C4	EMD Millipore	MABS487
Anti-FUS (*Xenopus*)	Abcam	ab70381

**Bacterial and Virus Strains**		

*E. coli* BL21(DE3)	New England Biolabs	C25271

**Chemicals, Peptides, and Recombinant Proteins**		

Thioflavin T	Sigma	2390-54-7
pFTAA	This manuscript	N/A
Puromycin	Sigma	P8833
AdOx (Adenosine-2′,3′-dialdehyde)	Sigma	N/A
Bovine chymotrypsin	Promega, Fitchburg, WI, USA	N/A
AcTEV Protease	ThermoFisher Scientific	12575015
ULP protease	Purified in the lab	N?A
PAD Cocktail, Active	SignalChem	P312-37C

**Critical Commercial Assays**		

mMessage mMachine SP6 Transcription Kit	ThermoFisher Scientific	AM1340
polyadenylated using Poly(A)-tailing kit	ThermoFisher Scientific	AM1350

**Deposited Data**		

Raw image data	Mendeley Data	https://doi.org/10.17632/4mjh8y579j.1

**Experimental Models: Cell Lines**		

Human: SH-SY5Y neuroblastoma cells	ATCC	CRL-2266
Sf9 cells	ThermoFisher Scientific	N/A
DH10EMBacY	Geneva Biotech	N/A

**Experimental Models: Organisms/Strains**		

*Xenopus laevis*	Nasco	LM00715MX

**Recombinant DNA**		

pACEBac2 vector	Geneva Biotech	N/A
pOPINS vector	This lab	N/A
mCherry-TAF	Table S1	N/A
mCherry-EWS	Table S1	N/A
mCherry-Transportin-1	Table S1	N/A
FUS	Table S1	N/A
FUS 6R→K	Table S1	N/A
FUS 6R→A	Table S1	N/A
FUS +16R	Table S1	N/A
FUS +16R ncY→A	Table S1	N/A
FUS +21R	Table S1	N/A
FUS ncY→F	Table S1	N/A
FUS ncY→A	Table S1	N/A
LC	Table S1	N/A
CTD	Table S1	N/A

**Software and Algorithms**		

Proteome Discoverer version 2.1.0.81 software	Thermo Scientific	N/A
PEAKS Studio version 8	Bioinformatics Solutions Incorporated, Waterloo, ON, Canada	N/A
ImageJ 1.50i (Java 1.8.0_131 (32-bit))	Wayne Rasband, NIH, USA	https://imagej.nih.gov/ij/
Zen 2.3 SP1 Black (v14.0.0.201)	Carl Zeiss Microscopy Gmbh	RRID:SCR_013672
MATLAB	Mathworks	N/A
Volocity	PerkinElmer	N/A
GraphPad Prism	GraphPad Software, Inc	N/A

**Other**		

μ-slides glass bottomed chambers	Ibidi GmbH, Germany	N/A
ZnSe windows	Platypus Technologies, USA	N/A

### Contact for Reagent and Resource Sharing

Further information and requests for resources and reagents should be directed to and will be fulfilled by the Lead Contact, Peter St George-Hyslop (phs22@cam.ac.uk and p.hyslop@utoronto.ca).

### Experimental Model Details

#### Cell lines

SHSY-5Y cells were cultured in DMEM high glucose medium (Sigma) supplemented with 10% FCS and 100 units/mL of penicillin and 100 μg/mL of streptomycin in a humidified incubator at 37°C and 5% CO2. SH-SY5Y cells stably expressing EYFP-FUS were generated by electroporation, followed by selection with geneticin. Cells were transiently transfected with plasmids of EYFP-FUS, FUS mutants, mCherry, mCherry-TAF, mCherry-EWS, and mCherry-TNPO1 using lipofectamine 3000 (Thermo Fisher Scientific) according to the manufacturer’s instructions. AdOx (Adenosine-2′,3′-dialdehyde, Sigma), or equal volume of DMSO vehicle, was added to cells for 24 hours at a final concentration of 20μM, unless otherwise stated in the figure legends.

#### *Xenopus* embryonic retina culture

*Xenopus laevis* embryos were fertilized *in vitro* and raised in 0.1x Modified Barth’s Saline at 18°C. Capped mRNAs of mCherry, mCherry-TNPO1 or FUS-GFP were synthesized using mMessage mMachine SP6 Transcription Kit (ThermoFisher Scientific), polyadenylated using Poly(A)-tailing kit (ThermoFisher Scientific), and injected into the two dorsal blastomeres at four-cell stage as described (Leung and Holt, 2008). Eye primordia from stage 34 embryos were dissected and cultured in 60% L15 on laminin-coated coverslips at 20°C for 24 hours. This research has been regulated under the Animals (Scientific Procedures) Act 1986 Amendment Regulations 2012 following ethical review by the University of Cambridge Animal Welfare and Ethical Review Body (AWERB).

### Method Details

#### Expression and purification of FUS TNPO1, EWS and TAF15

Constructs encoding FUS residues 1-526 and its mutants, LC-mEmerald (aa1-214) and CTF-mCherry (aa215-526), were cloned into pACEBac2 vector with a TEV cleavable N-terminal MBP tag and an EmGFP or mCherry-6xHis- C-terminal tag. Proteins were expressed and purified from insect Sf9 cells infected with the baculovirus. After four days of infection cells were harvested by spinning at 4000rpm for 30 minutes. Cell pellets were mixed with the resuspension buffer containing 50 mM Tris, 1 M KCl, 0.1% CHAPS, 1 mM DTT, 5% glycerol at pH 7.4, and proteins purified using three steps purification scheme including, Ni-NTA affinity column, Amylose affinity column followed by size exclusion chromatography in the buffer containing 50 mM Tris, 1 M KCl, 1 mM DTT, 5% glycerol at pH 7.4. For Thioflavin T binding experiment, FUS samples were produced without the C-terminal GFP tag.

Constructs encoding full length human EWS or human TAF15 were cloned into pBACEBac2 vector with a TEV protease cleavable N-terminal MBP tag. Proteins were expressed in Sf9 cells and purified on an amylose column. Fusion proteins were subjected to TEV protease cleavage and the MBP tag was further removed by size exclusion chromatography.

Gene encoding TNPO1 protein was cloned into pOPINS vector containing an N-terminal His-Sumo Tag and a ULP protease cleavage site separating the tag from TNPO1. Protein was expressed in *E. coli* BL21(DE3) in an overnight TB autoinduction media at 37°C for 5 hours followed by an overnight incubation at 25°C. Cells were harvested and subjected to lysis using high pressure cell disruption system. Clarified lysate was loaded on a Ni-NTA column and purified using standard procedure. Protein containing fractions were pooled, and dialysed in 25mM HEPES pH 7.5, 50mM NaCl, 1mM DTT and 5% glycerol buffer after addition of ULP protease to remove the His-Sumo Tag. Protein was further purified on a size-exclusion column and the fractions containing purified protein were pooled for all subsequent experiments.

For seeding experiments, a construct encoding full length FUS protein was cloned into pOPINS vector with an N-terminal His6-SUMO tag. Protein was expressed in *E. coli* BL21(DE3) using TB autoinduction medium, with induction at 25°C for 24 hours. Cells were lysed by sonication and proteins purified on Ni-NTA resin, followed by buffer exchange step into 50 mM Tris, 500 mM NaCl, pH 7.4.

#### Protein modification of FUS

For the preparation of hypomethylated FUS samples, the insect cell cultures were subjected to a repeated dose of 25μM AdOx solubilised in DMSO for four days. Protein was subjected to same methods of purification as unmodified FUS.

For the preparation of citrullinated FUS sample, 20μg of Protein arginine deiminases (PAD) cocktail comprising five (PAD1-5) full length GST tagged recombinant proteins was mixed with prewashed 50μl of anti-GST agarose bead in PAD cocktail buffer (0.1M Tris, 10mM CaCl_2_, pH 7.4). After incubating at 4°C for 1 hour, the sample was spun, supernatant was discarded and the beads were washed three times with PAD buffer before being incubated with 100μg of purified Em-GFP- FUS protein in 100μl of PAD buffer plus 200mM NaCl. The mixture was incubated at 37°C shaking for 2 hours. Post incubation the sample was centrifuged and the supernatant containing the citrullinated protein was collected and kept at 4°C until further analysis.

#### Immunoprecipitation

AdOx treated YFP-FUS expressing SHSY-5Y cells were lysed in RIPA buffer (50 mM Tris-HCl, pH 7.4, 150 mM NaCl, 0.25% sodium deoxycholate, 1% NP-40, 1 mM EDTA) with Protease and Phosphatase Inhibitor cocktail (Pierce) and 1mM PMSF for 20 minutes on ice. Supernatants were cleared by centrifugation and equal amounts of protein taken for total lysate input or for immunoprecipitation (IP) with anti-FUS antibody or normal mouse IgG control, followed by incubation with Protein G-Agarose (GE Healthcare) or Dynabeads Protein G (Invitrogen). Beads were washed four times with RIPA buffer before the addition of 1x LDS sample buffer (Thermo) containing 2.5 mM DTT (LSD/DTT), and samples analyzed by standard immunoblotting techniques. Protein expression was quantified using densitometry analysis with ImageJ software, and the amount of ADMA-FUS, normalized to FUS input, was expressed relative to control treated protein levels.

#### RIPA Insoluble FUS

Equal numbers of AdOx or DMSO treated YFP-FUS expressing cells were lysed directly in 1x LDS/DTT for input control, or in RIPA buffer as performed for IP. Lysates were cleared of insoluble material by centrifugation (16,000 xg) at 4°C, the RIPA soluble supernatant diluted in 1x LDS/DTT, and the RIPA insoluble pellet washed again in RIPA buffer, re-centrifuged and suspended in 1x LDS/DTT. All samples were denatured by heated at 95°C, and analyzed by immunoblotting.

#### FUS Droplet Assay

FUS purified proteins (0.5 μM-2 μM) were subjected to a series of NaCl concentration (40mM-500mM) in a total volume of 20 μL. Samples were deposited on 8-well glass bottom Ibidi slides, incubated at room temperature for 10 minutes before being imaged on a Zeiss Axiovert 200M microscope with Improvision Openlab software using 100X magnification objective. Droplet formation was followed over time by collecting a series of images in both the bright field and FITC channels. ImageJ software was used in all image processing. For all purified proteins n ≥ 3.

#### LC-mEmerald and CTF-mCherry co-operative mixing experiments

Purified LC-mEmerald and CTF-mCherry were buffer exchanged to a buffer containing 150mM NaCl. 1μM each were mixed and the concentration of the NaCl dropped to 50mM to induce droplet formation.

#### Turbidity Assay

2μM FUS protein was mixed with various NaCl concentrations in a 50 μL total volume in a Greiner 96 well half-are clear microplate. Sample were incubated at room temperature for 10 minutes prior to the absorption (turbidity) measurement at 600nm in a SpectraMax microplate reader. Readings were recorded in triplicate for each protein sample. All assays were performed in triplicate (n = 3).

#### Thioflavin Binding

Thioflavin (ThT) binding was evaluated by monitoring ThT fluorescence. The ThT solution, containing 10μM of ThT in 50mM Tris, 40mM NaCl (pH 7.4) buffer, was mixed with 2μM of control treated FUS, 2μM AdOx treated FUS and 5μM Synuclein and incubated for 15 minutes at room temperature. Fluorescence emission spectra of ThT, excited at 446 nm, were recorded between 455 and 550 nm on a PerkinElmer LS55 luminescence spectrometer using excitation and emission bandwidths of 2.5 nm. All binding experiments were carried out in triplicate (n = 3).

#### Circular dichroism

Circular dichroism (CD) spectrum of ADMA FUS, HYPO FUS, FUS +9R, FUS +16R, FUS +21R, FUS ncY→A, FUS ncY→F were measured on a JASCO-810 Spectropolarimeter at 25°C. 5μM of each purified protein was placed in a 1 mm path length quartz cuvette and the far-UV spectrum recorded in the wavelength range of 195 – 250 nm. Scans were repeated ten times and then averaged to yield a final spectrum for each construct.

#### pFTAA binding

Cells expressing FUS were imaged on μ-slides glass bottomed chambers (ibidi GmbH, Germany). Pentameric formyl thiophene acetic acid (pFTAA) was used at a concentration of 300nM diluted in PBS for cellular assays.

To image FUS in solution, borosilicate glass coverslips (VWR international, 22 × 22 mm, product number 631-0124) were cleaned using an argon plasma cleaner (PDC-002, Harrick Plasma) for at least 1 h to remove any fluorescent residues. Prior to use, each batch of cover-slides were tested for fluorescent artifacts. FUS was diluted at a concentration of 1 μM in low salt buffer, adding pFTAA at a final concentration of 300nM. A drop of 8 μL of this mixture was placed over a coverslip and another coverslip was placed on top.

Imaging was performed using a 60x Plan Apo TIRF, NA 1.45 oil objective, (Nikon Corporation) mounted on an Eclipse TE2000-U microscope (Nikon Corporation) fitted with a Perfect Focus unit. Fluorescence was collected by the same objective was separated from the returning beam by a dichroic (Di01-405/488/532/635, Semrock), and passed through an emission filter (FF03-525/50-25, Semrock) for both CFP and pFTAA signals. Cells expressing CFP-FUS were excited with a 405nm laser (LBX-405-50-CIR-PP, Oxxius), while pFTAA was excited with a 488 nm laser (0488-06-01-0060-100, Cobolt MLD). The excitation power was 25 W/cm^2^ for both lasers measured in epifluorescence mode. Cells were imaged in epifluorescence mode, while protein in solution was imaged in HiLo. The images were recorded on an EMCCD camera (Evolve 512, Photometrics) operating in frame transfer mode (EMGain of 6.8 e−/ADU and 250 ADU/photon). Each pixel was 241 nm in length. The microscope was controlled with Micromanager software, and bursts of images were recorded at 20 frames per second. Each analyzed image corresponds to an average of 50 images.

In the experiments mixing FUS with TNPO1, the untagged FUS assemblies were mixed with an equimolar concentration of TNPO1, and the same NaCl and pFTAA concentrations and imaging paradigms. The pFTAA-positive assemblies were automatically counted using an ImageJ (NIH) macro with the function Find Maxima, and statistics were computed with MATLAB (Mathworks) and GraphPad Prism.

#### Mass spectrometry and relative quantitation of FUS methylation

Samples of purified Sf9-cell expressed MBP-FUS-mEmerald from AdOx-treated and untreated cultures, totalling 100 μg of protein each, were separately incubated at 60°C for 30 minutes with 20 mM Tris(2-carboxyethyl)phosphine. Subsequently, sample solutions were adjusted to 7.45 M urea and 45 mM 4-vinylpridine before being incubated in the dark for one hour. The denatured and reduced protein samples were concentrated by centrifugation within Microcon YM-30 filter cartridges (EMD Millipore, Billerica, MA, USA). The filters embedded in these cartridges were washed with 300μl of 10 mM triethylammonium bicarbonate (TEAB) then covered in 50 μL of 10 mM TEAB containing 2 μg of bovine chymotrypsin (for methylation analysis) or porcine trypsin (for citrullination analysis) (Promega, Fitchburg, WI, USA) and shaken for five minutes at 600 rpm.Proteolytic digestion was carried out at 37°C for 14 hours.

Next, peptides were collected by passing the digest and an additional 50 μl volume of 0.5 M NaCl through the cartridges. Chymotryptic digests were divided for unlabeled and iTRAQ analyses. One aliquot of each digest was diluted with 20 μl of 1 M TEAB before addition of iTRAQ 8plex reagent in 210 μL of ethanol (Sciex, Concord, ON, Canada). iTRAQ reaction mixtures were left for 2 hours then concentrated in a centrifugal evaporator at 36°C before being diluted in 100 μL of 0.1% formic acid in water then combined. The samples were acidified with formic acid then desalted using Bond Elut OMIX C18 pipette tips (Agilent, Santa Clara, California USA) according to the manufacturer’s instructions. Desalted samples were concentrated in a centrifugal evaporator at 36°C, diluted in 0.1% formic acid, and analyzed on an Easy-nLC 1000-Orbitrap Fusion system (Thermo Scientific, Waltham, MA, USA). Mass spectra were collected over 60-minute-long HPLC runs, during which the acetonitrile content of the mobile phase was increased from 0 to 30% (v/v) over 40 minutes, then to 99.9% over 10 minutes and finally held at 99.9% acetonitrile for 10 minutes. The two mobile phases used were water and acetonitrile, each with 0.1% formic acid (v/v). The flow rate of the HPLC system was 300 nl/min and the Acclaim PepMap RSLC (Thermo Scientific) analytical column used was 25 cm long with a 75 μm internal diameter, packed with 2 μm C18 particles having 100 Å pores.

All mass spectra were collected in positive ion mode with a nanoflow electrospray ionization source potential of 2200 V and an ion transfer tube temperature of 275°C. The data acquisition cycle for the iTRAQ analysis was 3 s long, beginning with an orbitrap precursor ion scan from m/z 400 to 2,000 at a resolution of 30,000 followed by MS^2^ scans and MS^3^ scans of the most abundant precursor ions and their dissociation products respectively. The MS^3^ spectra were collected at a resolution of 30,000. Ions detected in any survey scan having charge states less than 2 or greater than 6 or having intensities under 10,000 counts or that had been subjected to MS^2^ four times in the preceding 20 s of analysis were excluded from MS^2^ and MS^3^. Instrument parameters used in the analysis of unlabelled samples were identical to those used in the iTRAQ analysis, except MS^3^ scans were omitted.

Peptide sequencing and quantification from the LC-MS data were performed on Proteome Discoverer version 2.1.0.81 software (Thermo Scientific) with peptide-to-spectrum matches produced by Sequest HT. Cleavage specificity was set to the C-termini of phenylalanine, leucine, tryptophan and tyrosine residues with up to two missed cleavages allowed. Precursor and product ion mass tolerance were 20 ppm and 0.4 Da respectively. The FASTA protein database used for the search included all human canonical and isoform entries (Uniprot version Dec 20, 2015, downloaded Mar 1, 2016), all Uniprot annotated *Spodoptera frugiperda* entries (downloaded Mar 13, 2017), bovine chymotrypsin (Uniprot accessions P00766, P00767, Q7M3E1), the small ubiquitin-like modifier of *Saccharomyces cerevisiae* (Uniprot accession Q12306), as well as the sequence of the MBP-FUS-mEmerald construct. False discovery rate estimation was performed using Percolator and quantification was undertaken with Reporter Ion Quantifier algorithm, both under default settings. The mass spectra were also interpreted using PEAKS Studio version 8 (Bioinformatics Solutions Incorporated, Waterloo, ON, Canada) with default settings.

#### AFM-IR

Analysis by conventional Atomic Force Microscopy and nanoIR2 (Anasys Instrument, USA) was performed on hydrophobic ZnSe windows (Platypus Technologies, USA). An aliquot of 10 μl of each sample was deposited on the surface for 1 minute. Successively, the droplet was rinsed by 1 mL of Milli-Q water and dried by a gentle stream of nitrogen.

A nanoIR2 platform (Anasys, USA), which combines high resolution and low noise AFM with a tunable OPO laser with top illumination configuration was used. The samples morphology was scanned by the nanoIR microscopy system, with a rate line within 0.05-0.2 Hz and in contact mode. A silicon gold coated PR-EX-nIR2 (Anasys, USA) cantilever with a nominal radius of 30 nm and an elastic constant of about 0.2 N/m was used. In order to avoid and reduce polarization effects, because of the gold coating of the tip, IR light was polarized parallel to the surface of deposition. All images were acquired with a resolution between 800x200 and 1000x500 pixels per line. The AFM images were treated and analyzed using SPIP software. The height images were first order flattened, while IR and stiffness related maps where only flattened by a zero order algorithm (offset). Average frequency shift, related to the intrinsic stiffness of the sample (Volpatti et al., 2016), was calculated on 3 WT, 4 hypo-methylated (HM) and 4 FUS +16R droplets. The last ones could be divided into different regions according to their relative stiffness. Relative stiffness was calculated as the normalized ratio of the average frequency shift of each region. The relative values were measured at both the 1^st^ and 2^nd^ resonance of oscillation of the cantilever with consistent results.

The spectra were collected by placing the AFM tip on the top of the FUS droplets (Figure S1) with a laser wavelength sampling of 2 cm^-1^ with a spectral resolution of 4 cm^-1^ and 256 co-averages, within the range 1400-1800 cm^-1^ (Müller et al., 2014).

Within a droplet, several spectra at different positions were acquired. For each droplet, an average spectrum was obtained as the average of the spectra at different positions within its area and by subtracting the baseline signal of the substrate. Successively, they were smoothed by Savitzky-Golay filter (second order, 9 points) and normalized. Spectra second derivatives were calculated and smoothed by Savitzky-Golay filter (second order, 9 points)). In total, 55 spectra were acquired for three methylated FUS droplets, 73 spectra for four hypo-methylated assemblies and 88 for four FUS +16R droplets. Relative secondary and quaternary organization was evaluating integrating the area of the different secondary structural contribution in the amide band I. Spectra were analyzed using the microscope’s built-in Analysis Studio (Anasys) and OriginPRO. All measurements were performed at room temperature and with laser power between 1%–4% of the maximal one and under controlled Nitrogen atmosphere with residual real humidity below 5%.

#### Quantification of cells with FUS granule clusters

For live cells, images were taken immediately after 24 h mock and AdOx treatment. For fixed cell quantification, cells were initially fixed with 4% PFA in medium at 37°C for 15 min and then with 4% PFA in PBS at room temperature for 15 min. Hoechst or DAPI was used for nuclear counter-staining. Images were taken using a Zeiss 710 confocal microscope. For transient transfected cells, AdOx was added 3 hours before transfection. All the experiments were replicated at least three times. To quantify the percentage of cells with FUS granule clusters, more than 250 cells were counted for each sample.

#### Immunocytochemistry, puromycin labeling and imaging in retinal cultures

For immunocytochemistry detecting endogenous FUS proteins, heat-induced antigen retrieval in sodium citrate buffer (10mM sodium citrate, 0.05% Tween 20, pH 6.0) was performed after 4% paraformaldehyde/15% sucrose fixation. Cultures were subsequently permeabilized with 0.1% Triton (Sigma) for 5 minutes, blocked in 5% heat-inactivated goat serum and incubated at 4°C overnight with anti-FUS antibody (Abcam, ab70381). For puromycin labeling, axons pre-treated with DMSO or 20μM AdOx for 30 minutes were severed from their cell bodies and subsequently incubated with 10μg/ml puromycin (Sigma) for 10 minutes. After fixation, permeablisation and blocking steps, puromycin-incorporated nascent peptides were labeled with Alexa Fluor 647-conjugated anti-puromycin antibody (1:250, Millipore) overnight. 161 Randomly selected noncollapsed growth cones in each condition from 3 independent biological replicates were imaged using a Nikon Optiphot inverted microscope equipped with a 60x oil-immersion objective and a CCD camera (Hamamatsu).

For quantitation of fluorescence intensity, the growth cone outline was traced on the phase contrast image using Volocity (PerkinElmer), then superimposed on the fluorescent image. The software calculated the fluorescent intensity within the growth cone, giving a measurement of pixel intensity per unit area. The growth cone outline was then placed in an adjacent area clear of cellular material to record the background fluorescent intensity. This reading was subtracted from the growth cone reading, yielding the background-corrected intensity. All quantitative analysis was performed ‘blind’ to experimental condition and normalized to the control.

FUS-GFP fluorescence in live distal axon segments was imaged under a Perkin Elmer Spinning Disk UltraVIEW ERS, Olympus IX81 inverted microscope with a 60x 1.4NA silicone oil objective, equipped with a Flash4.0 camera (Hamamatsu). Images were acquired at maximum speed for 1 minute with 500ms exposure time.

### Quantification and Statistical Analyses

In all figures the mean and SEM are described along with the number of biological replications, and the statistical tests applied.

### Data and Software Availability

Raw image data of full western blots are deposited in Mendeley Data at https://doi.org/10.17632/4mjh8y579j.1.

## Supplemental Information

Table S1. Listing of the FUS Expression Constructs Used in This Study, Related to the Key Resources Table Listing by name and mutation architecture of the FUS expression constructs used in this study.

Video S1. Structured Illumination Microscopy Movies of Representative Single-Droplet Collisions for ADMA FUS, Related to Figure 1E Structured illumination microscopy videos of representative single droplet collisions for ADMA FUS at 0 ms, 200 ms and 680 ms. ADMA FUS droplets fuse into a single larger droplet, confirming their liquid phase state. Scale bar = 2 μm.

Video S2. Structured Illumination Microscopy Images of Individual Droplet Collisions for HYPO FUS, Related to Figure 1E Structured illumination microscopy images of individual droplet collisions for HYPO FUS at 0 ms, 200 ms and 680 ms. HYPO FUS droplets collide but do not fuse, indicating that they either have high viscosity and high surface tensions or have converted to a gel-like state. Scale bar = 2 μm.

Video S3. Live Cell Imaging Reveals that mCherry-Tagged TNPO1 Exists in Motile Granules in Distal Axon Shafts, Related to Figure 7E Live cell imaging reveals that mCherry-tagged TNPO1 exists in motile granules in distal axon shafts. TNPO1 can be seen in granules moving bidirectionally over a 50 s interval. Scale bar = 10 μm.
