# Supplementary material for: FUS Phase Separation Is Modulated by a Molecular Chaperone and Methylation of Arginine Cation-π Interactions
Source: Cell. 2018 Apr 19;173(3):720–734.e15. doi: 10.1016/j.cell.2018.03.056 (PMC5927716; doi:10.1016/j.cell.2018.03.056)
Supplement: Table S1. Listing of the FUS Expression Constructs Used in This Study, Related to the Key Resources Table — Listing by name and mutation architecture of the FUS expression constructs used in this study. [file mmc1.docx]

**TABLE S1**

| ***Common name of FUS expression construct*** | ***Mutations*** |
| --- | --- |
| FUS-FL-WT-EmGFP | WT |
| FUS-FL-WT-mCherry | WT |
|  |  |
| FUS 6R 🡪 A | R216A, R259A, R407A, R472A, R473A, R476A |
| FUS 6R 🡪 K | R216K, R259K, R407K, R472K, R473K, R476K |
|  |  |
| FUS +5R | G167R, G170R, G173R, G189R, G191R |
| FUS +7R | G167R, G170R, G173R, G189R, G191R, G202R, S205R |
| FUS +9R | G167R, G170R, G173R, G202R, S205R, S221R, G225R, G228R, M254R |
| FUS +16R | G167R, G170R, G173R, G202R, S205R, S221R, G225R, G228R, G230R, M254R, G379R, N381G, insert387G, G398R, G401R, S402G, G404R, G456R, M464R |
| FUS +21R | G167R, G170R, G173R, G189R, G191R, G202R, S205R, S221R, G225R, G228R, G230R, M254R, G380R, N381G, M392R, G398R, G401R, S402G, G404R, G414R, G417R, G456R, M464R |
|  |  |
| FUS ncY🡪A | Y113A, Y122A, Y130A, Y136A, Y143A, Y149A, Y161A |
| FUS ncY🡪F | Y113F, Y122F, Y130F, Y136F, Y143F, Y149F, Y155F, Y161F |
| FUS + 16R ncY🡪A | Y113A, Y122A, Y130A, Y136A, Y143A, Y149A, Y161A, G167R, G170R, G173R, G202R, S205R, S221R, G225R, G228R, G230R, M254R, G379R, N381G, insert387G, G398R, G401R, S402G, G404R, G456R, M464R |
|  |  |
| LC-EmGFP | aa 1-214 |
| CTD-mCherry | aa 215-526 |
